# Threshold-free high-power methods for the ontological analysis of genome-wide gene-expression studies

**DOI:** 10.1186/gb-2007-8-5-r74

**Published:** 2007-05-08

**Authors:** Björn Nilsson, Petra Håkansson, Mikael Johansson, Sven Nelander, Thoas Fioretos

**Affiliations:** 1 Department of Clinical Genetics, Lund University Hospital, SE-221 85 Lund, Sweden; 2 Department of Automatic Control, Royal Institute of Technology, SE-100 44 Stockholm, Sweden; 3 Computational Biology Center, Memorial Sloan-Kettering Cancer Center, New York, NY 10021, USA

## Abstract

Ontological analysis facilitates the interpretation of microarray data. Here we describe new ontological analysis methods which, unlike existing approaches, are threshold-free and statistically powerful. We perform extensive evaluations and introduce a new concept, detection spectra, to characterize methods. We show that different ontological analysis methods exhibit distinct detection spectra, and that it is critical to account for this diversity. Our results argue strongly against the continued use of existing methods, and provide directions towards an enhanced approach.

## Abstract 2

New ontological analysis methods are described for microarray data interpretation that, unlike existing approaches, are threshold-free and statistically powerful.

## Background

A fundamental challenge in genome-wide gene-expression studies is to translate complex microarray data into an understanding of the biological conditions being studied. A widely used approach to this problem is ontological analysis - or functional gene-category analysis - the aim of which is to enable data interpretation in the light of known functional relationships between genes. In essence, the methodology seeks to identify categories of functionally associated genes - predefined in external ontologies such as the Gene Ontology Consortium taxonomy [1] - that show deviating expression patterns compared to the general gene population. The underlying motivation is that such categories are presumably likelier to be biologically relevant than gene categories whose expression patterns do not exhibit distinctive features.

Most ontological analysis approaches published so far rely on discrete statistical procedures (binomial, hypergeometric, chi-square or Fisher's exact test) to test for relative enrichments of gene categories within lists of significant genes [2]. These methods are widely used and numerous software packages exist. Nevertheless, discrete methods suffer from a drawback in that the results fundamentally depend on an (essentially arbitrary) threshold for calling genes differentially or non-differentially expressed [3,4].

To overcome this problem, threshold-free methods for identifying potentially relevant gene categories were recently proposed. Most of these are based on the Kolmogorov-Smirnov (KS) goodness-of-fit test [3-6], although rank-based approaches have also been suggested [7,8]. The important conceptual advantage of threshold freedom is that the expression data for all genes are considered simultaneously, without the uncertainty associated with previous gene list extraction.

In the study reported here, we enhance the ontological analysis methodology in several important respects. Particularly, we first consider enhanced methods for detecting potentially relevant gene categories. These methods are based on classical and recent examples of a particular class of goodness-of-fit techniques - empirical distribution function (EDF) statistics - that are threshold-free and can be expected to have high statistical power: that is, the chance of detecting a relevant gene category, given it is there, is increased. We carefully assess each method using extensive simulations and by application to multiple real microarray datasets. Second, we develop a new concept, 'detection spectra', which serves to map the prototypic gene categories that are preferentially detected by a given method. We show that different ontological analysis methods exhibit distinct detection spectra, and that it is critical to be aware of this diversity. We also show that, in terms of detection spectrum, the methods represent a continuum ranging from KS on the one extreme to the discrete methods on the other, whereas the remaining methods exhibit intermediate properties. In particular, one method based on the Zhang C (ZC) statistic qualifies as an effective, threshold-free replacement for discrete methods, something that has been previously lacking. Third, to simplify the characterization of detected categories in terms of underlying enrichments of over- or underexpressed genes, we equip each method with an indicator function. These functions indicate the direction of transcriptional deviation, and support the biological interpretation of the ontological analysis results. Finally, we develop a fast significance computation scheme that allows EDF-based analyses to be performed in acceptable time. In conclusion, we introduce attractive alternatives to existing methods for the ontological analysis of microarray experiments, and give directions for the choice of method in practice.

## Results

### Evaluation by simulation

**Figure 1:**
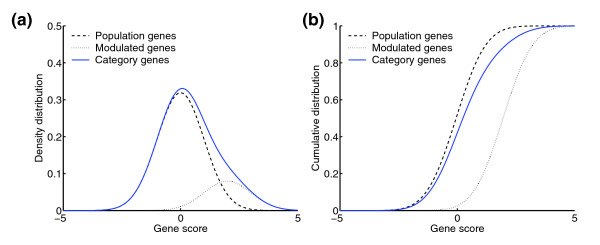
Simulation model used for artificially recreating gene categories with known expression pattern deviations. **(a) **Density distribution for the reference population (dashed black line), the modulated genes (dotted black line), and the resulting mixture (solid blue line). **(b) **Corresponding cumulative distributions. The parameter values used in this example were *π *= 0.2, *μ *= 1.0, and *σ *= 1.0, modeling a low-proportion admixture of moderately overexpressed genes.

We first performed an extensive series of simulations, carefully designed to systematically assess the ability of each method to detect gene categories with varying expression pattern deviations (details in Materials and methods). In short, we simulated the global gene population by drawing 10,000 gene scores from a standard normal distribution. To simulate gene categories with known deviations, we used a mixture model [4] in which a proportion of the genes are given scores from a modulated normal distribution whereas the remaining genes scores follow a standard normal distribution like the population (Figure 1). Four parameters control the types of categories modeled: the number of genes in the category (*N*); the proportion of modulated genes (*π*); and the mean and standard deviation of the modulated gene scores (*μ *and *σ*). By varying these parameters, we could artificially recreate gene categories with a broad range of score-distribution dissimilarities.

### Detection spectra

To achieve near-exhaustive testing, we selected 1,800 parameter configurations from wide and relevant intervals, and determined the method powers for each one (see Materials and methods). Hence, for each method, we obtain an 1,800-dimensional performance profile, or detection spectrum, indicating the category types that can be detected.

**Figure 2:**
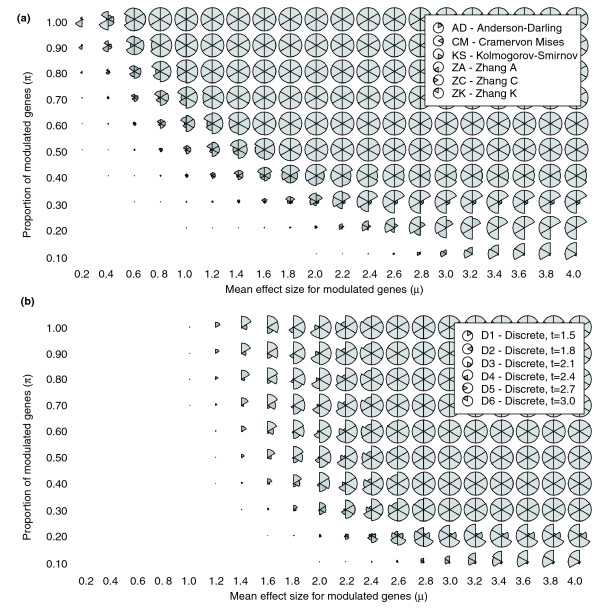
Detection spectra. **(a) **Partial detection spectra (*N *= 30, *σ *= 0.5; complete spectra in Additional data file 1; raw data in Additional data file 5) for the EDF-based methods. **(b) **Corresponding results for the discrete method with six different thresholds for calling genes differentially expressed (*t*). The sector radiuses are proportional to the parameter-specific powers. The mappings between sectors and methods are given in the in-figure legends. Key observations: (1) Different ontological analysis methods exhibit distinct detection spectra. (2) The threshold-free methods exhibit higher coverages than the discrete method, that is, they detect more diverse category types. (3) The discrete method, which is currently the most commonly used method, exhibits strong threshold-dependency, that is, it needs tweaking to yield good results. (4) Important differences are seen between the threshold-free methods (commented under Results).

We determined the detection spectra for the EDF-based methods and, for completeness, a discrete method with six thresholds for calling genes differentially expressed (D1 to D6; see Materials and methods). As evident in Figure 2 and Additional data file 1, the detection power varied considerably between methods and between category types: First, all methods worked well for detecting high-proportion-high-effects categories (Figure 2, upper right pie charts), but failed for low-proportion-low-effects categories (Figure 2, lower left pie charts). Second, all methods performed uniformly better in large than in small categories, owing to the fact that larger categories allow for detection of subtler deviations. Third, much more interestingly, substantial performance differences were observed for low to intermediate modulation effect sizes or proportions. In particular, some methods were better suited for detecting low-proportion-high-effects categories (Figure 2, lower right pie charts) whereas others were more apt for detecting high-proportion-low-effects categories (Figure 2, upper left pie charts). For low-proportion-high-effects categories, ZC and Zhang K (ZK) yielded the best results, followed by Zhang A (ZA) and Anderson-Darling (AD) (see Materials and methods). The discrete method also worked well, but exhibited strong threshold dependency. For high-proportion-low-effects categories, KS, the Cramér-von Mises (CM) statistic, and AD yielded the best results for narrow (*σ *= 0.1), intermediate (*σ *= 0.5) and diffuse (*σ *= 1.0) effect spreads, respectively. This is consistent with the fact that narrow effects spreads cause discrepancies near the center of the category gene score distribution (KS optimal), whereas intermediate and diffuse spreads lead to dissimilarities which, to greater extents, engage the tails (AD better suited). Fourth, we estimated the coverages of the detection spectra by computing overall (average) powers. The highest values were observed for AD, ZA, ZC, and ZK, implying that these methods are able to detect a broader range of categories than discrete and previous threshold-free (KS-based) methods (Additional data file 2). Taken together, these simulations clearly show that different ontological analysis methods focus on different types of categories, and provide an exact map of the method performances under varying circumstances.

### Method-method relationships

**Figure 3:**
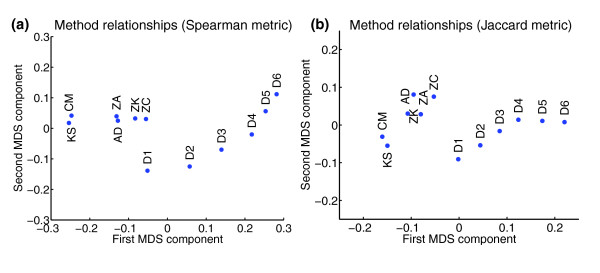
Method relationships. These are visualized using multidimensional scaling of the **(a) **Spearman and **(b) **Jaccard similarities (Additional data file 3). Proximate methods can be expected to yield similar category rankings (Spearman case) and sets of significant categories (Jaccard case). The figure shows that, property-wise, the methods range from KS and CM at the one extreme to the discrete method on the other (D1 to D6), whereas the other methods exhibit intermediate behaviors. Notably, ZC, with its strong ability to detect low-proportion-high-effects deviations, constitutes a threshold-free replacement for the discrete method. Method abbreviations are defined in Figure 2.

To gain an overview of the mutual method relationships, we next quantified the method-method agreements, that is, the expected concordances between results, by computing the Spearman and Jaccard metrics (see Materials and methods) for all pairs of category-detection statistics. Interestingly, multidimensional scaling (MDS) of the resulting similarity matrices (Additional data file 3) showed that, property-wise, the methods represent a continuum ranging from the high-proportion-low-effects-focused (that is, center oriented) KS and CM at the one extreme to the low-proportion-high-effects-focused (that is, tail oriented) discrete method on the other with the remaining methods in between (Figure 3). In particular, we observe that ZC is the closest threshold-free approximation to the discrete method, and, hence, should be regarded as an appealing replacement for that method. Furthermore, we note that ZK is only slightly less tail oriented than ZC, and that the pairs ZA versus AD, and KS versus CM yield similar results. Moreover, because MDS captures the largest variability in the data, Figure 3 shows that a major determining factor of detection spectrum diversity lies in the methods' preferences for detecting high-proportion-low-effects or low-proportion-high-effects categories. In conclusion, the agreement data summarize the method relationships, and provide directions for the choice of method in practice.

### Application to real data

We proceeded to apply all methods to real microarray data, starting with a dataset from a recent study of ours (P.H., B.N., A Andersson, C Lassen, U Gullberg, and T.F., unpublished work). The aim of this study was to map the transcriptional response of cells to the activity of the fusion oncogene *BCR/ABL1*, associated with chronic myeloid leukemia (CML), in a reverse way by blocking the activity of the fusion protein using the tyrosine-kinase inhibitory drug imatinib mesylate [9]. Essentially, expression profiles of imatinib-treated and non-treated CML cell lines were acquired, and gene scores quantifying the imatinib response were computed (see Materials and methods).

**Figure 4:**
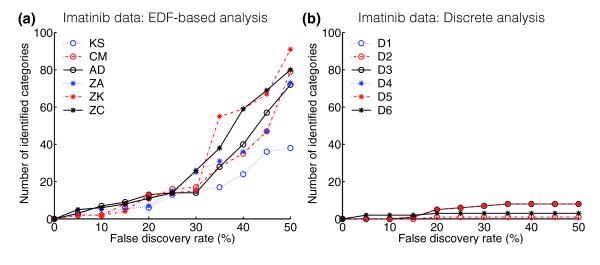
Application of the various methods to real data. **(a) **EDF methods;**(b) **discrete methods. The plots show the total numbers of categories detected (all six ontologies) in the imatinib data (P.H., B.N., A Andersson, C Lassen, U Gullberg, and T.F., unpublished work) at various false-discovery rates. Method abbreviations are defined in Figure 2.

**Figure 5:**
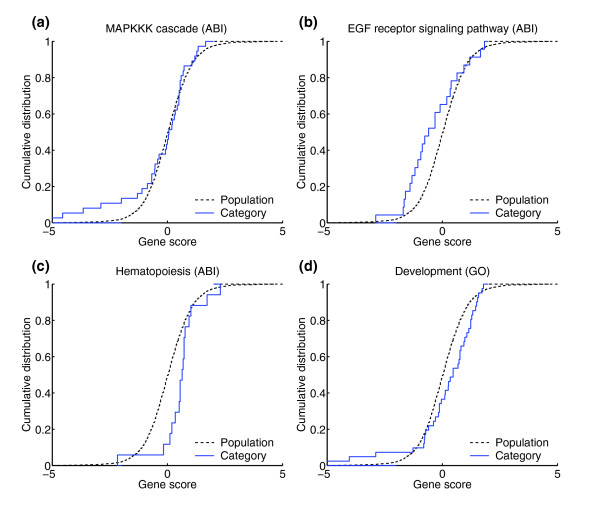
Links between distributions, detection spectra and indicator functions. To illustrate important connections between gene score distributions, detection spectra, and indicator functions, we selected four categories from the imatinib data. **(a) **The category **'**MAPKKK cascade' exhibits a heavy lower tail, exemplifying a low-proportion-high-effects enrichment of underexpressed genes. As expected, this category was detected by ZK and ZC. **(b) **The category 'EGF receptor signaling pathway' has normal tails but is left-shifted midway between the center and the tails, consistent with an intermediate-proportion-moderate-effects enrichment of underexpressed genes. This category was detected by AD. Whereas these two examples receive indicator values near -1 because they are enriched in underexpressed genes, category 'hematopoiesis' **(c) **exhibits a right-shifted distribution, implying indicator values near 1. **(d) **Category 'development', identified at a slightly higher false-discovery rate, has a heavy lower tail as well as a right-shifted center, and exemplifies mixed enrichments (intermediate AD, CM, ZA, and ZC indicator values).

As shown in Table 1, ontological analysis of the gene scores computed from the data from the imatinib experiment confirmed that the choice of method strongly influences the results when applied to real data. The overlaps between sets of detected categories approximately followed the simulations (Table 1), as did the category rankings (data not shown). Consistent with the overall power simulations, the threshold-free methods detected more categories than the discrete method, whereas the difference between the threshold-free methods were less pronounced (Figure 4). Investigating the putative biological relevance of the detected categories (Table 1), we noted several gene categories previously implicated in *BCR/ABL1*-mediated leukemogenesis or in the effects of imatinib. For example, consistent with data in the literature, significant enrichments of overexpression were observed in the categories 'heme biosynthesis', and enrichments of underexpression in the 'interferon-gamma signaling pathway', the 'MAPKKK cascade', and in categories related to apoptosis regulation. Also identified was the 'EGF receptor pathway', individual members of which are known to become phosphorylated/activated by *BCR/ABL1*. Taken together, these findings support the validity of the ontological analysis methodology. Finally, to point at important connections between gene score distributions and detection spectra, and to exemplify the utility of the indicator functions, we selected four illustrative categories, which are discussed in Figure 5.

**Table 1:** Functional profile of the imatinib-induced transcriptional response

KS	CM	AD	ZA	ZK	ZC	D1	D2	D3	D4	D5	D6	Δ_ZC_	Δ_ZK_	Size*	Biological process (GO)
+	+											-1.00	-1	3	'de novo' IMP biosynthesis
+	+	+	+	+	+	+	+	+	+	+		+1.00	+1	5	Heme biosynthesis
					+							-1.00	-1	3	Inactivation of MAPK activity
+	+											-1.00	-1	5	Intracellular transport
					+							-1.00	-1	5	Negative regulation of apoptosis
	+											-0.87	-1	22	Regulation of transcription
+	+											-0.97	-1	7	Regulation of translational initiation
+	+											-1.00	-1	7	Translational initiation

KS	CM	AD	ZA	ZK	ZC	D1	D2	D3	D4	D5	D6	Δ_ZC_	Δ_ZK_	Size*	Biological process (ABI)

+	+			+								+1.00	+1	17	Hematopoiesis
					+			+	+	+		-1.00	-1	25	Inhibition of apoptosis
				+	+			+	+	+		-0.89	-1	9	Macrophage-mediated immunity
				+	+			+	+	+		-0.99	-1	37	MAPKKK cascade
+	+	+		+								-1.00	-1	21	Protein complex assembly
				+								-1.00	-1	4	rRNA metabolism

KS	CM	AD	ZA	ZK	ZC	D1	D2	D3	D4	D5	D6	Δ_ZC_	Δ_ZK_	Size*	Molecular function (GO)

+	+	+	+									-0.90	-1	257	ATP binding
+												-0.61	-1	17	ATP-dependent helicase activity
+	+	+	+	+								-0.91	-1	57	GTPase activity
+	+	+	+	+								-1.00	-1	3	Protein kinase C activity
					+							-1.00	-1	6	Protein tyrosine phosphatase activity
+	+	+	+	+	+							-0.93	-1	95	RNA binding
+	+	+	+	+								-0.91	-1	14	Translation initiation factor activity

KS	CM	AD	ZA	ZK	ZC	D1	D2	D3	D4	D5	D6	Δ_ZC_	Δ_ZK_	Size*	Molecular function (ABI)

											+	-0.87	-1	18	Protein kinase

KS	CM	AD	ZA	ZK	ZC	D1	D2	D3	D4	D5	D6	Δ_ZC_	Δ_ZK_	Size*	Molecular pathway (ABI)

		+										-1.00	-1	23	EGR receptor signaling pathway
		+	+	+	+			+	+	+	+	-1.00	-1	8	Interferon-gamma signaling pathway
	+	+	+	+								-1.00	-1	3	Metabotropic glutamate receptor group I pathway

KS	CM	AD	ZA	ZK	ZC	D1	D2	D3	D4	D5	D6	Δ_ZC_	Δ_ZK_	Size*	Cellular component (GO)

	+	+	+		+							-1.00	-1	147	Cytoplasm
		+	+	+	+							+1.00	+1	4	Kinesin complex
			+	+	+			+	+	+	+	+0.73	+1	9	Microtubule associated complex
	+	+	+		+							-0.93	-1	23	Nuclear pore
		+	+		+							-1.00	-1	24	Nucleolus
			+	+								-1.93	-1	21	Ribonucleoprotein complex

Gene categories in the imatinib data (P.H., B.N., A Andersson, C Lassen, U Gullberg, and T.F., unpublished work) called significant by at least one of the category-detection methods (25% false-discovery rate; significance indicated by +). Key observations: (1) The choice of method strongly influences the results. (2) The method-method agreements observed on real data approximately follow those observed in the simulations (see Figure 3). (3) Several detected categories are consistent with literature data on BCR/ABL1-mediated leukemogenesis, supporting the validity of the methodology (see main text). (4) The table illustrates the use of indicator functions to determine the direction of transcriptional deviation in detected categories. In this case, ZC and ZK exemplify soft indicators (available for AD, CM, ZA, and ZC) and the less informative hard indicators (available for KS and ZK), respectively (see also Figure 5). *By size, we mean the number of unique genes (Entrez Gene IDs) within the category.

### Application to other studies

To verify the generality of our results, we applied all methods to 25 other differential expression comparisons (Additional data file 4) based on seven publicly available microarray datasets (see Materials and methods). While a detailed description of the vast amount of resulting data is beyond the scope of this paper, we point out the following recurrent observations. First, as in Table 1, the choice of method strongly impacted on the results in accordance with the simulated method-method agreements. Second, the numbers of detected categories also approximately followed the simulated method relationships. In broad outline, the threshold-free methods detected many more categories than the discrete methods, because of the noticeable difference in overall power between these two groups. In contrast, the differences within the threshold-free group were less pronounced and more variable, which is explained by the fact that these methods have more similar overall powers, implying that the number of detected categories will, to a greater extent, be determined by the match between the detection spectrum and the set of deviating categories that are actually present in the data. Taken together, these findings further underscore the fact that different methods focus on different category types, and, hence, that it is important to be aware of this in practice.

### Computational efficiency

The total time required for analyzing the 25 studies (all methods and ontologies) was 32 seconds (C++ implementation; 2 GHz Core2Duo PC), illustrating the benefit of the fast significance computation scheme (see Materials and methods).

## Discussion

The ontological analysis of genome-wide studies relies fundamentally on the validity and continued growth of ontologies providing annotations of gene function. However, efficient computational methods are needed to integrate these annotations with data in an optimal way. We have addressed the latter problem by considering gene-category identification methods based on high-power EDF statistics.

We have shown that the value of these methods lies in their higher overall powers - implying an ability to detect a broader range of potentially biologically relevant gene categories - and in their detection spectra, which are distinct from those of existing methods. Previously, KS-based and discrete approaches have focused on high-proportion-low-effects and low-proportion-high-effects deviations, respectively [4], whereas methods with intermediate detection spectra and threshold-free methods for detecting low-proportion-high-effects deviations have been lacking. The methods described fill these gaps. In particular, our data suggest ZC to be a new method of choice for low-proportion-high-effects-oriented analysis. Offering excellent low-proportion-high-effects coverage, high overall power and the obvious advantages of threshold freedom, ZC virtually removes the need for discrete methods. Regarding the remaining methods, ZK is slightly less tail oriented than ZC; ZA and AD focus on intermediate-proportion- moderate-effects categories; Finally, CM resembles KS.

As shown, the choice of category-detection method has a profound impact on the results of the ontological analysis. However, the question of what prototypic categories are most biologically relevant, and hence should be the primary target in ontological analyses, is an open problem. In the absence of solid evidence supporting that one category type is generally more biologically relevant than others, the choice of method must be partly guided by the investigator's preferences and project-specific considerations. For example, in data containing strongly differentially expressed genes (for example, the imatinib study presented here), it may be natural to optimize the analysis for low-proportion-high-effects categories, making the tail-sensitive methods (ZC, ZK and discrete) the methods of choice. On the other hand, in datasets where differentially expressed genes display predominantly low-to-moderate effect sizes, it seems more reasonable to focus on intermediate-proportion-moderate-effects and high-proportion-low-effects categories, motivating the choice of methods with more center-oriented detection spectra in this case.

Alternatively, multiple methods can be used in concert, provided that appropriate statistical corrections are made. Such an approach would yield results tables similar to Table 1, and offers the benefit of allowing the user to make indirect conclusions about the characteristics of the distributional deviations of the detected categories. For example, if a category is detected as significant by all methods (for example, the heme biosynthesis category in the imatinib experiment), then, quite clearly, its gene-score distribution must be highly aberrant, most probably because of a high-proportion-high-effects size enrichment. In contrast, if a category is called significant by one method (for example, AD with the EGF receptor signaling pathway in the imatinib data), and not by the others, then the distributional deviations must fall within the detection spectra of that method but outside the detection spectra of the other methods. In the EGF receptor signaling pathway example, a reasonable conclusion - given the detection spectra established in Additional data file 1 - would be that underlying deviation is likely to be of intermediate-proportion-moderate-effects-size type, as such categories represent the detection optimum of AD.

Regardless of category-detection statistic, the reference gene population, null model, gene score, and ontology also influence the results and must be chosen judiciously [2,3,7]. We recognize that a shared limitation of many ontological analysis methods, ours included, is that dependencies between genes are not taken into account when computing significances, something that may lead to underestimated *p *values. First steps have been taken to develop dependency-modeling schemes, for example SAFE [3] or CatMap [7]. While the methods described can be adopted into those frameworks if desired, additional efforts are needed to address the problem of modeling dependencies in detail.

Other features introduced are indicator functions and a fast significance computation scheme. The indicator functions facilitate the interpretation of the results of the ontological analysis. Their advantage compared to existing approaches is that the need for two separate tests per category, one to detect enrichment of overexpression and one to detect enrichment of underexpression, is removed. A limitation is that enrichments cannot be distinguished from (contralateral) depletions. Such ambiguities can be resolved graphically (Figure 5), or through the development of improved versions in future studies. The fast significance computations are not crucial to the ontological analysis as such, but are valuable in that they allow the procedure to be performed within an acceptable time-frame.

Finally, we recognize the limitations of the gene-category model used for computing the detection spectra. First, as already discussed, the model assumes independence between genes. Second, for tractability, we have limited our treatment to gene categories with only one group of the modulated genes. While the model could be extended to multiple modulated groups, this would obviously increase the complexity of the study at the expense of presentational clarity and understandability. Third, we have only considered gene scores with approximately normal distributions, which is a minor limitation as the most frequently used gene scores are based on *t*-statistics. Nevertheless, the properties of the described methods for scores with distinctly different distributions (for example, scores based on the *F*-statistic) remain to be established.

## Conclusion

We have presented novel ontological analysis methods constituting attractive alternatives to existing approaches. Hence, this work contributes to the repertoire of useful methods aiding the interpretation of genome-wide gene expression studies.

## Materials and methods

### Threshold-free category-detection methods

Let *F*_*N*_(*x*) and *F*(*x*) denote the empirical (cumulative) distribution functions for the gene-specific differential expression scores *x*_1_,...,*x*_*N *_for an *N*-gene category and the scores *x*'_1_,...,*x*'_*M *_for an *M*-gene reference gene population, respectively. We consider six EDF statistics to measure discrepancy between *F*_*N*_(*x*) and *F*(*x*), that is, to detect gene categories with deviating gene-score distributions. A technicality that arises is that, normally when using EDF statistics, *F*(*x*) is specified by a continuous function. This is obviously not the case here as *F*(*x*) is an EDF, jumping by 1/*M *at each *x*'_*i*_. However, we note that, in this application, this issue can be ignored because *M *is large (on the order of 5,000 to 40,000), making *F*(*x*) sufficiently smooth to be regarded as continuous.

First, we consider the Kolmogorov-Smirnov (KS) statistic [10,11], which is, without doubt, the best-known EDF statistic and, as stated above, has been used previously for ontological analysis. The KS statistic is the largest distance between *F*(*x*) and *F*_*N*_(*x*)

D=x∈Rsup⁡|FN(x)−F(x)|=i=1...Nmax⁡max⁡{iN−yi,yi−i−1N},
 MathType@MTEF@5@5@+=feaafiart1ev1aaatCvAUfeBSjuyZL2yd9gzLbvyNv2Caerbhv2BYDwAHbqedmvETj2BSbqee0evGueE0jxyaibaiKI8=vI8tuQ8FMI8Gi=hEeeu0xXdbba9frFj0=OqFfea0dXdd9vqai=hGuQ8kuc9pgc9s8qqaq=dirpe0xb9q8qiLsFr0=vr0=vr0dc8meaabaqaciGacaGaaeqabaqadeqadaaakqaaeeqaaiaadseacqGH9aqpdaqhaaWcbaGaamiEaiabgIGiolaadkfaaeaaciGGZbGaaiyDaiaacchaaaGccaGG8bGaamOramaaBaaaleaacaWGobaabeaakiaacIcacaWG4bGaaiykaiabgkHiTiaadAeacaGGOaGaamiEaiaacMcacaGG8baabaGaeyypa0Zaa0baaSqaaiaadMgacqGH9aqpcaaIXaGaaiOlaiaac6cacaGGUaGaamOtaaqaaiGac2gacaGGHbGaaiiEaaaakiGac2gacaGGHbGaaiiEaiaacUhadaWcaaqaaiaadMgaaeaacaWGobaaaiabgkHiTiaadMhadaWgaaWcbaGaamyAaaqabaGccaGGSaGaamyEamaaBaaaleaacaWGPbaabeaakiabgkHiTmaalaaabaGaamyAaiabgkHiTiaaigdaaeaacaWGobaaaiaac2hacaGGSaaaaaa@60F8@

where *y*_*i *_= *F*(*x*_*i*_). While intuitively straightforward and capable of detecting discrepancies near the center of the distribution, KS fails to notice subtle discrepancies in the tails as well as small but consistent deviations. Second and third, we use two members of the Cramér-von Mises family of quadratic EDF statistics, defined by

N∫−∞∞(FN(x)−F(x))2ψ(x)dF(x),
 MathType@MTEF@5@5@+=feaafiart1ev1aqatCvAUfeBSjuyZL2yd9gzLbvyNv2Caerbhv2BYDwAHbqedmvETj2BSbqee0evGueE0jxyaibaiKI8=vI8tuQ8FMI8Gi=hEeeu0xXdbba9frFj0=OqFfea0dXdd9vqai=hGuQ8kuc9pgc9s8qqaq=dirpe0xb9q8qiLsFr0=vr0=vr0dc8meaabaqaciGacaGaaeqabaqadeqadaaakeaacaWGobWaa8qmaeaacaGGOaGaamOramaaBaaaleaacaWGobaabeaakiaacIcacaWG4bGaaiykaiabgkHiTiaadAeacaGGOaGaamiEaiaacMcacaGGPaWaaWbaaSqabeaacaaIYaaaaaqaaGGaciab=jHiTGGaaiab+5HiLcqaaiab+5HiLcqdcqGHRiI8aOGaeqiYdKNaaiikaiaadIhacaGGPaGaamizaiaadAeacaGGOaGaamiEaiaacMcacaGGSaaaaa@4D4F@

where *ψ*(*x*) is a suitable weight function. We consider *ψ*(*x*) = 1, which generates the Cramér-von Mises (CM) statistic itself [12]

W2=N∫−∞∞(FN(x)−F(x))2dF(x)=112N+∑i=1N(yi−2i−12N)2,
 MathType@MTEF@5@5@+=feaafiart1ev1aqatCvAUfeBSjuyZL2yd9gzLbvyNv2Caerbhv2BYDwAHbqedmvETj2BSbqee0evGueE0jxyaibaiKI8=vI8tuQ8FMI8Gi=hEeeu0xXdbba9frFj0=OqFfea0dXdd9vqai=hGuQ8kuc9pgc9s8qqaq=dirpe0xb9q8qiLsFr0=vr0=vr0dc8meaabaqaciGacaGaaeqabaqadeqadaaakqaaeeqaaiaadEfadaahaaWcbeqaaiaaikdaaaGccqGH9aqpcaWGobWaa8qmaeaacaGGOaGaamOramaaBaaaleaacaWGobaabeaakiaacIcacaWG4bGaaiykaiabgkHiTiaadAeacaGGOaGaamiEaiaacMcacaGGPaWaaWbaaSqabeaacaaIYaaaaaqaaiabgkHiTiabg6HiLcqaaiabg6HiLcqdcqGHRiI8aOGaamizaiaadAeacaGGOaGaamiEaiaacMcaaeaacqGH9aqpdaWcaaqaaiaaigdaaeaacaaIXaGaaGOmaiaad6eaaaGaey4kaSYaaabCaeaacaGGOaGaamyEamaaBaaaleaacaWGPbaabeaakiabgkHiTmaalaaabaGaaGOmaiaadMgacqGHsislcaaIXaaabaGaaGOmaiaad6eaaaaaleaacaWGPbGaeyypa0JaaGymaaqaaiaad6eaa0GaeyyeIuoakiaacMcadaahaaWcbeqaaiaaikdaaaGccaGGSaaaaaa@6122@

which is able to integrate small consistent deviations, regarded as more powerful than KS, but is still not optimal for detecting discrepancies in the tails. Therefore, we also consider *ψ*(*x*) = *F*(*x*)^-1^(1-*F*(*x*))^-1 ^which gives more weight to the tails and generates the Anderson-Darling (AD) statistic [13]

A2=N∫−∞∞(FN(x)−F(x))2F(x)(1−F(x))dF=−N−1N∑i=1N(2i−1)(ln⁡yi+ln⁡(1−yN+1−i)).
 MathType@MTEF@5@5@+=feaafiart1ev1aqatCvAUfeBSjuyZL2yd9gzLbvyNv2Caerbhv2BYDwAHbqedmvETj2BSbqee0evGueE0jxyaibaiKI8=vI8tuQ8FMI8Gi=hEeeu0xXdbba9frFj0=OqFfea0dXdd9vqai=hGuQ8kuc9pgc9s8qqaq=dirpe0xb9q8qiLsFr0=vr0=vr0dc8meaabaqaciGacaGaaeqabaqadeqadaaakqaaeeqaaiaadgeadaahaaWcbeqaaiaaikdaaaGccqGH9aqpcaWGobWaa8qmaeaadaWcaaqaaiaacIcacaWGgbWaaSbaaSqaaiaad6eaaeqaaOGaaiikaiaadIhacaGGPaGaeyOeI0IaamOraiaacIcacaWG4bGaaiykaiaacMcadaahaaWcbeqaaiaaikdaaaaakeaacaWGgbGaaiikaiaadIhacaGGPaGaaiikaiaaigdacqGHsislcaWGgbGaaiikaiaadIhacaGGPaGaaiykaaaaaSqaaiabgkHiTiabg6HiLcqaaiabg6HiLcqdcqGHRiI8aOGaamizaiaadAeaaeaacqGH9aqpcqGHsislcaWGobGaeyOeI0YaaSaaaeaacaaIXaaabaGaamOtaaaadaaeWbqaaiaacIcacaaIYaGaamyAaiabgkHiTiaaigdacaGGPaGaaiikaiGacYgacaGGUbGaamyEamaaBaaaleaacaWGPbaabeaakiabgUcaRiGacYgacaGGUbGaaiikaiaaigdacqGHsislcaWG5bWaaSbaaSqaaiaad6eacqGHRaWkcaaIXaGaeyOeI0IaamyAaaqabaGccaGGPaGaaiykaiaac6caaSqaaiaadMgacqGH9aqpcaaIXaaabaGaamOtaaqdcqGHris5aaaaaa@736E@

Fourth, fifth, and sixth, we include three EDF statistics recently derived by Zhang [14]. These are denoted Zhang A (ZA), Zhang C (ZC) and Zhang K (ZK) to reflect theoretical relationships with AD, CM, and KS. However, simulations have shown that the Zhang statistics are sometimes substantially more powerful [14]. The derivations of the Zhang statistics are beyond the scope of this paper, but can be found in the original work. The computing formulas are:

ZA=−∑i=1N(ln⁡yiN−i+1/2+ln⁡(1−yi)i−1/2),ZC=∑i=1Nln⁡2(yi−1−1(N−1/2)/(i−3/4)−1),ZK=i=1...Nmax⁡((i−12)ln⁡(i−1/2Nyi)+(N−i+12)ln⁡(N−i+1/2N(1−yi))).
 MathType@MTEF@5@5@+=feaafiart1ev1aaatCvAUfeBSjuyZL2yd9gzLbvyNv2Caerbhv2BYDwAHbqedmvETj2BSbqee0evGueE0jxyaibaiKI8=vI8tuQ8FMI8Gi=hEeeu0xXdbba9frFj0=OqFfea0dXdd9vqai=hGuQ8kuc9pgc9s8qqaq=dirpe0xb9q8qiLsFr0=vr0=vr0dc8meaabaqaciGacaGaaeqabaqadeqadaaakqaabeqaaiaadQfadaWgaaWcbaGaamyqaaqabaGccqGH9aqpcqGHsisldaaeWbqaaiaacIcadaWcaaqaaiGacYgacaGGUbGaamyEamaaBaaaleaacaWGPbaabeaaaOqaaiaad6eacqGHsislcaWGPbGaey4kaSIaaGymaiaac+cacaaIYaaaaiabgUcaRmaalaaabaGaciiBaiaac6gacaGGOaGaaGymaiabgkHiTiaadMhadaWgaaWcbaGaamyAaaqabaGccaGGPaaabaGaamyAaiabgkHiTiaaigdacaGGVaGaaGOmaaaaaSqaaiaadMgacqGH9aqpcaaIXaaabaGaamOtaaqdcqGHris5aOGaaiykaiaacYcaaeaacaWGAbWaaSbaaSqaaiaadoeaaeqaaOGaeyypa0ZaaabCaeaaciGGSbGaaiOBamaaCaaaleqabaGaaGOmaaaakiaacIcadaWcaaqaaiaadMhadaqhaaWcbaGaamyAaaqaaiabgkHiTiaaigdaaaGccqGHsislcaaIXaaabaGaaiikaiaad6eacqGHsislcaaIXaGaai4laiaaikdacaGGPaGaai4laiaacIcacaWGPbGaeyOeI0IaaG4maiaac+cacaaI0aGaaiykaiabgkHiTiaaigdaaaaaleaacaWGPbGaeyypa0JaaGymaaqaaiaad6eaa0GaeyyeIuoakiaacMcacaGGSaaabaGaamOwamaaBaaaleaacaWGlbaabeaakiabg2da9maaDaaaleaacaWGPbGaeyypa0JaaGymaiaac6cacaGGUaGaaiOlaiaad6eaaeaaciGGTbGaaiyyaiaacIhaaaGccaGGOaGaaiikaiaadMgacqGHsisldaWcaaqaaiaaigdaaeaacaaIYaaaaiaacMcaciGGSbGaaiOBaiaacIcadaWcaaqaaiaadMgacqGHsislcaaIXaGaai4laiaaikdaaeaacaWGobGaamyEamaaBaaaleaacaWGPbaabeaaaaGccaGGPaGaey4kaSIaaiikaiaad6eacqGHsislcaWGPbGaey4kaSYaaSaaaeaacaaIXaaabaGaaGOmaaaacaGGPaGaciiBaiaac6gacaGGOaWaaSaaaeaacaWGobGaeyOeI0IaamyAaiabgUcaRiaaigdacaGGVaGaaGOmaaqaaiaad6eacaGGOaGaaGymaiabgkHiTiaadMhadaWgaaWcbaGaamyAaaqabaGccaGGPaaaaiaacMcacaGGPaGaaiOlaaaaaa@A7A6@

### Discrete category detection methods

For comparison, we also included a discrete category-detection method in the study. As a representative of this class of methods, we used the binomial test, which is routinely used as an approximation to hypergeometric procedures, such as Fisher's exact test, when the population is large. The technical details can be found in standard statistics textbooks or in work on ontological analysis (see [2] and references therein). Throughout, we used six thresholds for calling genes differentially expressed (1.5, 1.8 to 3.0; denoted D1 to D6) and used gene scores that would always be compatible with these values (see Microarray datasets).

### Category characterization methods

While the EDF statistics effectively detect deviating - and thus presumably biologically relevant - gene categories, they do not, in their basic form, indicate whether the deviations are caused by enrichments of overexpressed genes, underexpressed genes, or a mixture of both. In previous approaches, this problem has been addressed by performing two separate tests for each category, one to detect enrichments of overexpression and one to detect enrichments of underexpression. Here, we proceed differently and instead derive an indicator for each detection method. The advantage of these functions is that the direction of transcriptional deviation is determined in a continuous manner, removing the need for double testing.

In the case of CM, AD, ZA, and ZC, these statistics can be readily rewritten as ∑i=1Nδi
 MathType@MTEF@5@5@+=feaafiart1ev1aqatCvAUfeBSjuyZL2yd9gzLbvyNv2Caerbhv2BYDwAHbqedmvETj2BSbqee0evGueE0jxyaibaiKI8=vI8tuQ8FMI8Gi=hEeeu0xXdbba9frFj0=OqFfea0dXdd9vqai=hGuQ8kuc9pgc9s8qqaq=dirpe0xb9q8qiLsFr0=vr0=vr0dc8meaabaqaciGacaGaaeqabaqadeqadaaakeaadaaeWaqaaiabes7aKnaaBaaaleaacaWGPbaabeaaaeaacaWGPbGaeyypa0JaaGymaaqaaiaad6eaa0GaeyyeIuoaaaa@3B66@, where *δ*_*i *_are defined separately for each statistic and depend on *x*_*i *_and *y*_*i *_(calculations not shown). We let the indicators Δ be the raw correlation between δ={δi}1N
 MathType@MTEF@5@5@+=feaafiart1ev1aqatCvAUfeBSjuyZL2yd9gzLbvyNv2Caerbhv2BYDwAHbqedmvETj2BSbqee0evGueE0jxyaibaiKI8=vI8tuQ8FMI8Gi=hEeeu0xXdbba9frFj0=OqFfea0dXdd9vqai=hGuQ8kuc9pgc9s8qqaq=dirpe0xb9q8qiLsFr0=vr0=vr0dc8meaabaqaciGacaGaaeqabaqadeqadaaakeaacqaH0oazcqGH9aqpcaGG7bGaeqiTdq2aaSbaaSqaaiaadMgaaeqaaOGaaiyFamaaDaaaleaacaaIXaaabaGaamOtaaaaaaa@3C5D@ and δ′={sign(FN(xi)−F(xi))⋅δi}1N
 MathType@MTEF@5@5@+=feaafiart1ev1aqatCvAUfeBSjuyZL2yd9gzLbvyNv2Caerbhv2BYDwAHbqedmvETj2BSbqee0evGueE0jxyaibaiKI8=vI8tuQ8FMI8Gi=hEeeu0xXdbba9frFj0=OqFfea0dXdd9vqai=hGuQ8kuc9pgc9s8qqaq=dirpe0xb9q8qiLsFr0=vr0=vr0dc8meaabaqaciGacaGaaeqabaqadeqadaaakeaacuaH0oazgaqbaiabg2da9iaacUharuavP1wzZbItLDhis9wBH5gaiqaacaWFZbGaa8xAaiaa=DgacaWFUbGaaiikaiaadAeadaWgaaWcbaGaamOtaaqabaGccaGGOaGaamiEamaaBaaaleaacaWGPbaabeaakiaacMcacqGHsislcaWGgbGaaiikaiaadIhadaWgaaWcbaGaamyAaaqabaGccaGGPaGaaiykaiabgwSixlabes7aKnaaBaaaleaacaWGPbaabeaakiaac2hadaqhaaWcbaGaaGymaaqaaiaad6eaaaaaaa@53CC@, that is,

△=<δ,δ′>‖δ‖2‖δ′‖2=∑i=1Nδi2⋅sign(FN(xi)−F(xi))∑i=1Nδi2
 MathType@MTEF@5@5@+=feaafiart1ev1aaatCvAUfeBSjuyZL2yd9gzLbvyNv2Caerbhv2BYDwAHbqedmvETj2BSbqee0evGueE0jxyaibaiKI8=vI8tuQ8FMI8Gi=hEeeu0xXdbba9frFj0=OqFfea0dXdd9vqai=hGuQ8kuc9pgc9s8qqaq=dirpe0xb9q8qiLsFr0=vr0=vr0dc8meaabaqaciGacaGaaeqabaqadeqadaaakeaacqWIZwIvcqGH9aqpdaWcaaqaaiabgYda8iabes7aKjaacYcacuaH0oazgaqbaiabg6da+aqaamaafmaabaGaeqiTdqgacaGLjWUaayPcSdWaaSbaaSqaaiaaikdaaeqaaOWaauWaaeaacuaH0oazgaqbaaGaayzcSlaawQa7amaaBaaaleaacaaIYaaabeaaaaGccqGH9aqpdaWcaaqaamaaqadabaGaeqiTdq2aa0baaSqaaiaadMgaaeaacaaIYaaaaOGaeyyXICncbaGaa83Caiaa=LgacaWFNbGaa8NBaiaacIcacaWGgbWaaSbaaSqaaiaad6eaaeqaaOGaaiikaiaadIhadaWgaaWcbaGaamyAaaqabaGccaGGPaGaeyOeI0IaamOraiaacIcacaWG4bWaaSbaaSqaaiaadMgaaeqaaOGaaiykaiaacMcaaSqaaiaadMgacqGH9aqpcaaIXaaabaGaamOtaaqdcqGHris5aaGcbaWaaabmaeaacqaH0oazdaqhaaWcbaGaamyAaaqaaiaaikdaaaaabaGaamyAaiabg2da9iaaigdaaeaacaWGobaaniabggHiLdaaaaaa@6C9C@

When the denominator is zero, we let Δ = 0. For KS and ZK, which are based on max operators instead of sums, we let

Δ = *sign*(*F*_*N*_(*x*_*i*_) - *F*(*x*_*i*_)),

where *i *is the index used when computing the KS or ZK statistic. The Δ indicators characterize gene categories by considering the distributional dissimilarities that led to their detection. In categories with unexpectedly many overexpressed genes, we have *F*_*N*_(*x*_*i*_) ≤ *F*(*x*_*i*_) for all *i*, implying Δ = 1. Conversely, categories with unexpectedly many underexpressed genes, will receive Δ = -1. Moreover, for CM, AD, ZA and ZC, Δ will attain intermediate values depending on the balance between the two types of genes. The KS and ZK indicators are less informative, evaluating to either -1 or 1 depending on the predominant direction of deviation.

### Significance computations

The null distributions for the EDF statistics are unknown, and, in some cases (ZA, ZC and ZK), asymptotic theory is lacking. To compute significances, we therefore used a procedure based on Monte Carlo simulation by gene permutations, which is currently a standard scheme in ontological analysis although it does not account for dependencies between genes. More elaborate schemes seeking to model dependencies using sample label permutations have been suggested [3,7], and the methods above can be adopted into those frameworks if needed.

In principle, the null distributions could be simulated from scratch for every category. However, that approach turned out to be exceedingly time-consuming. We instead note that the assumed continuity of *F*(*x*) implies that the EDF statistics are distribution-free. Hence, their null distributions can be pre-computed by drawing *y*_*i*_'s from a uniform distribution, a procedure that is essentially equivalent to permuting genes when the population is large (assumed). This strategy completely avoids simulations at runtime, allowing entire ontological analyses to be performed in instants. Throughout, the distributions were pre-simulated using 10^8 ^Monte Carlo replicates (per category size and statistic), and were compressed to tractable sizes using a recent algorithm (B.N. unpublished work).

### Simulation model

To simulate the reference gene score population, we drew 10,000 scores from a standard normal distribution (zero mean, unit variance). This choice is motivated by the fact that differential expression is frequently assessed using the *t*-statistic or variance-moderated versions thereof [15-17], in which cases the population scores will be approximately normally distributed as most genes are non-differentially expressed. Furthermore, to simulate deviating gene categories, we used the mixture model previously proposed in [4], in which a proportion of the category genes are given scores from a modulated normal distribution (non-zero mean, non-unit variance) whereas the remaining genes are given scores from a standard normal like the reference population (Figure 1). The model parameters are: the number of category genes (*N*), the proportion of modulated genes (*π*), the mean (effect size) of the modulated gene scores (*μ*), and the standard error (effects spread) of the modulated gene scores (*σ*). The parameter values were: *N *= 10, 30 and 100 genes, which are typical category sizes; *π *= 0.1, 0.2 to 1.0, which is essentially exhaustive; *μ *= 0.2, 0.4 to 4.0, covering very weak to very strong effects. Because the EDF statistics are distribution-symmetric, negative and positive *μ *values will yield identical results. Hence, the evaluation can be restricted to positive values without loss of generality. Finally, *σ *= 0.1, 0.5 and 1.0, corresponding to narrow, intermediate and diffuse effects spreads, respectively. Thus, the total number of four-parameter combinations was 3 × 10 × 20 × 3 = 1,800. For each combination, 100,000 random categories were generated and tested for conformity with the population distribution. The parameter-configuration-specific statistical powers were estimated as the proportions of categories called significant at the *p *< 0.001 level (the full set of raw data is in Additional data file 5). To verify robustness, the experiments were repeated with numerous other cutoff levels, yielding results in broad agreement with those presented.

To quantify the diversity of category types detected, we computed overall (average) powers across all parameter configurations and across *π *and *μ *for fixed *N *and *σ*. To quantify method-method agreements, we computed the Spearman rank correlation and the Jaccard similarity coefficient (or Jaccard index) for all pairs of methods. The Spearman metric, the correlation between the rank-transformed *p *values, measures similarity between category rankings. The Jaccard similarity coefficient, the proportion of categories called significant by both methods, reflects similarity between sets of detected categories.

### Microarray datasets

The imatinib data (P.H., B.N., A Andersson, C Lassen, U Gullberg, and T.F. unpublished work) were generated at our lab by culturing five CML cell lines in the presence or absence of imatinib mesylate. Expression profiles were obtained at 3 and 12 h after drug exposure using 27 K cDNA arrays. For each time point and treatment group, two technical replicates were obtained, yielding 2 × 2 × 2 = 8 arrays per cell line. After filtering and probe merging, 5,532 unique Entrez Gene entries remained. The full set of microarray data will be made available upon acceptance of the original work.

In addition to the dataset from the imatinib experiment, we included seven publicly available expression array datasets. The Valk dataset [18] and the Radich dataset [19] were obtained from the NCBI Gene Expression Omnibus repository [20], accessions GSE1159 and GSE4170, respectively. The Zheng dataset [21] was obtained from the ArrayExpress repository [22]. The Bhattacharjee dataset [23] was obtained from the Broad Institute website [24]. The Ross dataset [25] was obtained from the St Jude Children's Research Hospital website [26]. The Andersson dataset [27] was obtained by personal communication with the corresponding author. The West dataset [28] was obtained from the Duke University website [29].

As a score of differential expression, we used Smyth's moderated *t*-statistic [15], which follows an approximate *t*-distribution under the null hypothesis whenever the data are reasonably normal. Hence, in any given study, the variance of the population scores will be near one, guaranteeing that the thresholds used with the discrete method are meaningful.

### Ontologies

A total of six ontologies from the Gene Ontology (GO) Consortium [30] and the Applied Biosystems Panther Gene Classification System (ABI) [31] were used: Biological Process (GO+ABI), Molecular Function (GO+ABI), Cellular Component (GO), and Molecular Pathway (ABI). The ontology versions used in the analyses were those available in December 2006.

### Software availability

To allow readers to readily apply the described methods to their own data, we provide a software package called RenderCat (stand-alone Windows executable). This software is publicly and freely available on request from B.N. The source code is open and can be downloaded from the SourceForge repository [32]. The package includes implementations of all the category-detection methods described, including the indicator functions and the fast significance computations. For the convenience of the user, we have also included functionality for creating tables similar to Table 1 (tab-delimited text or LaTeX format) and capability for rendering gene-category score-distribution plots similar to Figure 5 (bitmap format). To correct for multiple testing, the program uses the false-discovery rate [33,34].

## Additional data files

Additional data are available online with this paper. Additional data file 1 is a figure representing the complete results of the simulation study. Additional data file 2 is a table listing overall powers. Additional data file 3 is a table containing the complete data from the method-method agreement assessment study. Additional data file 4 contains a list of additional differential expression studies. Additional data file 5 contains the raw data used for generating the detection spectra.

## Supplementary Material

Additional data file 1 A figure representing the complete results of the simulation study. Click here for file

Additional data file 2 A table listing overall powers. Click here for file

Additional data file 3 A table containing the complete data from the method-method agreement assessment study. Click here for file

Additional data file 4 A list of additional differential expression studies. Click here for file

Additional data file 5 The raw data used for generating the detection spectra. Click here for file
