# Supplementary material for: Threshold-free high-power methods for the ontological analysis of genome-wide gene-expression studies
Source: Genome Biol. 2007 May 8;8(5):R74. doi: 10.1186/gb-2007-8-5-r74 (PMC1929143; doi:10.1186/gb-2007-8-5-r74)

Supplementary Figure

These figures represent the complete results for the simulation study. In the pie-charts, the sector radiuses are proportional to the statistical powers of the methods, *i.e.* the proportions of categories of the given type that were successfully detected. The mappings between sectors and methods are given in the in-figure legends. To facilitate reading, the results have been grouped such that each page shows the results for all values of π and μ fixed N and σ. Within each page, the top and bottom panels illustrate the results for the EDF-based methods and the discrete method, respectively. The x and y axes indicate the mean effect size for the modulated genes μ and the proportion of modulated genes π, respectively.

Detection spectrums and overall powers: 10-gene categories, $\sigma = 0.1$

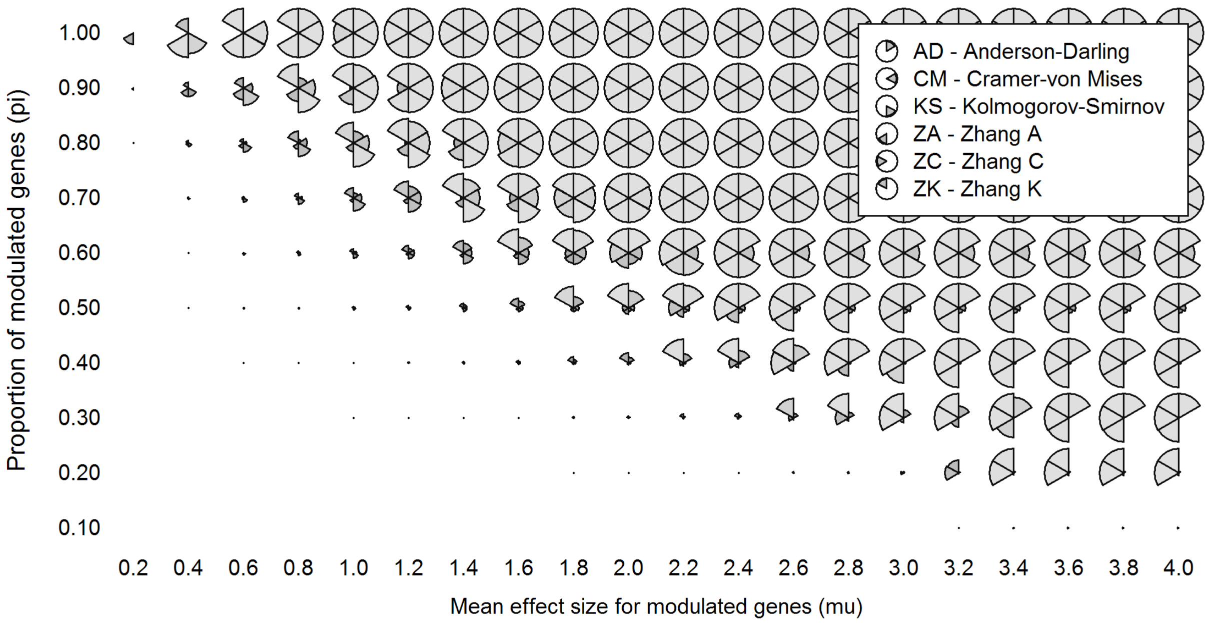

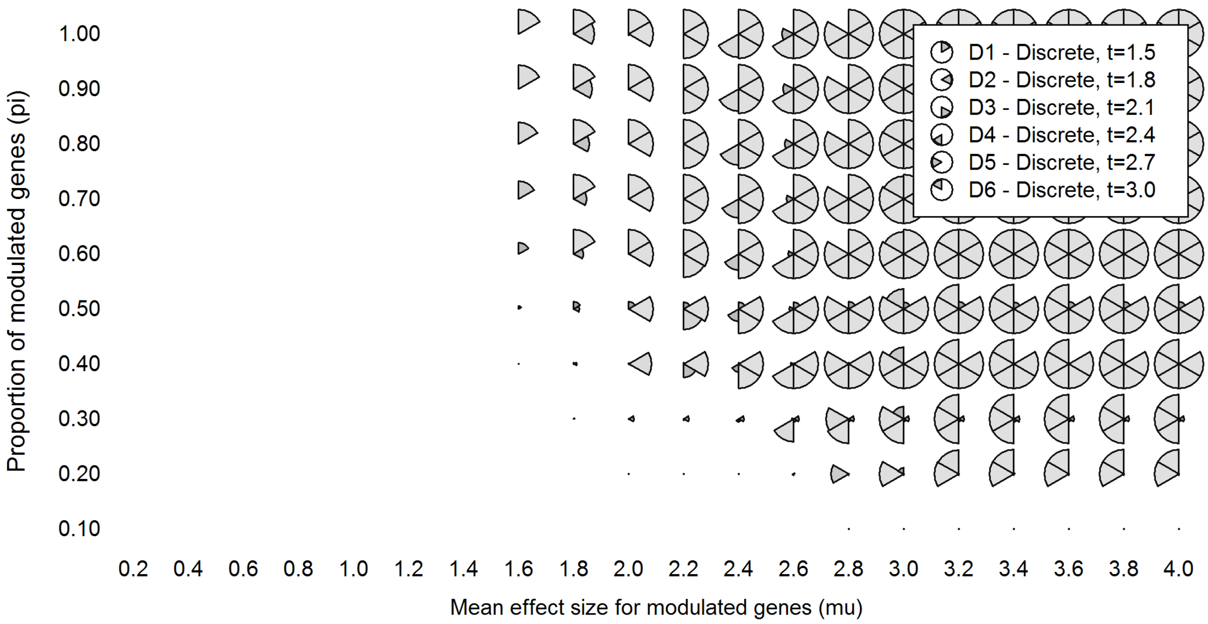

Detection spectrums and overall powers: 30-gene categories, σ=0.1

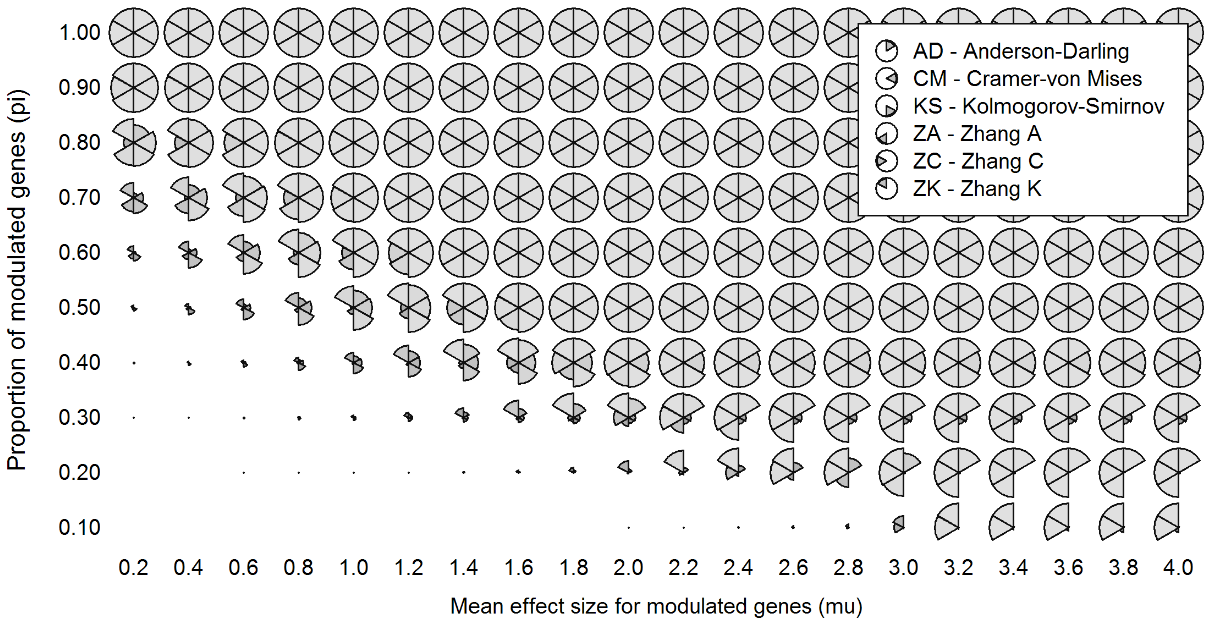

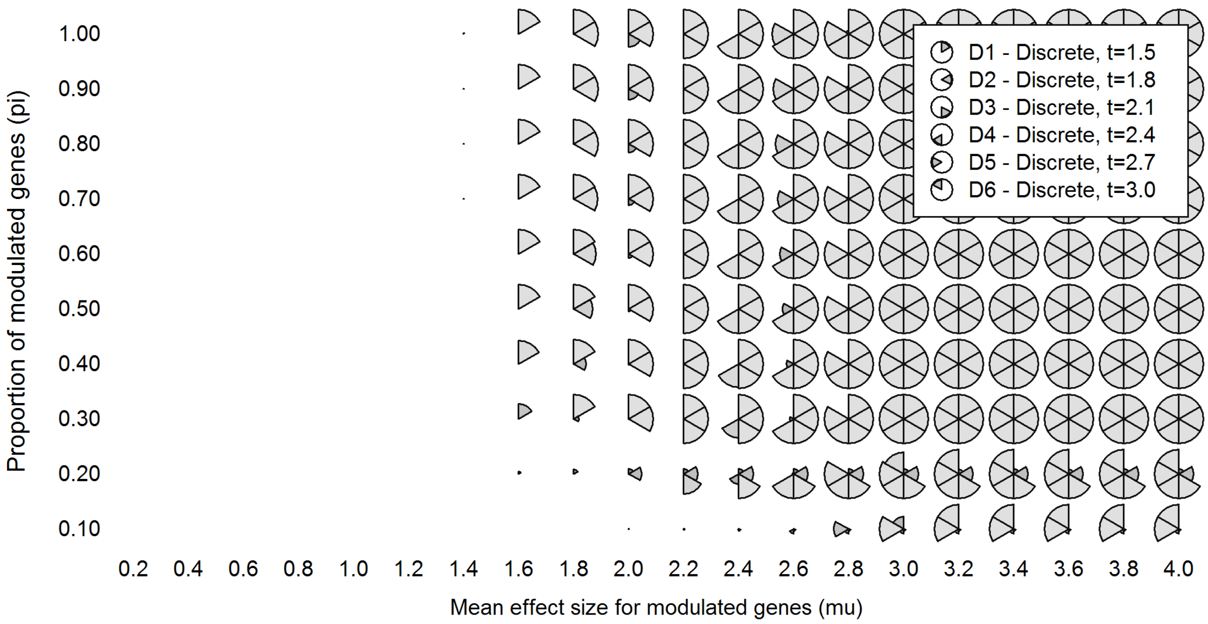

Detection spectrums and overall powers: 100-gene categories, σ=0.1

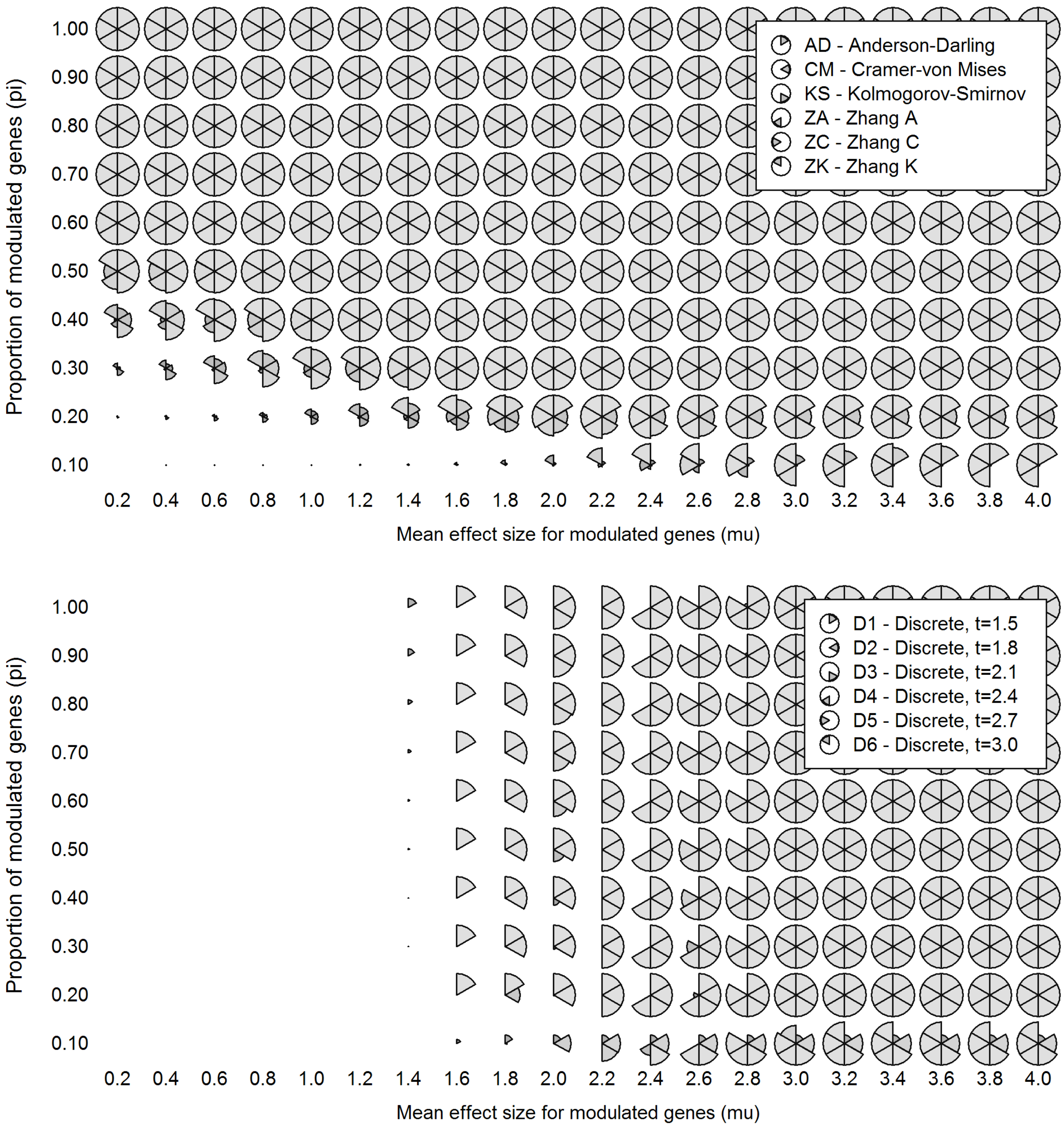

Detection spectrums and overall powers: 10-gene categories, $\sigma = 0.5$

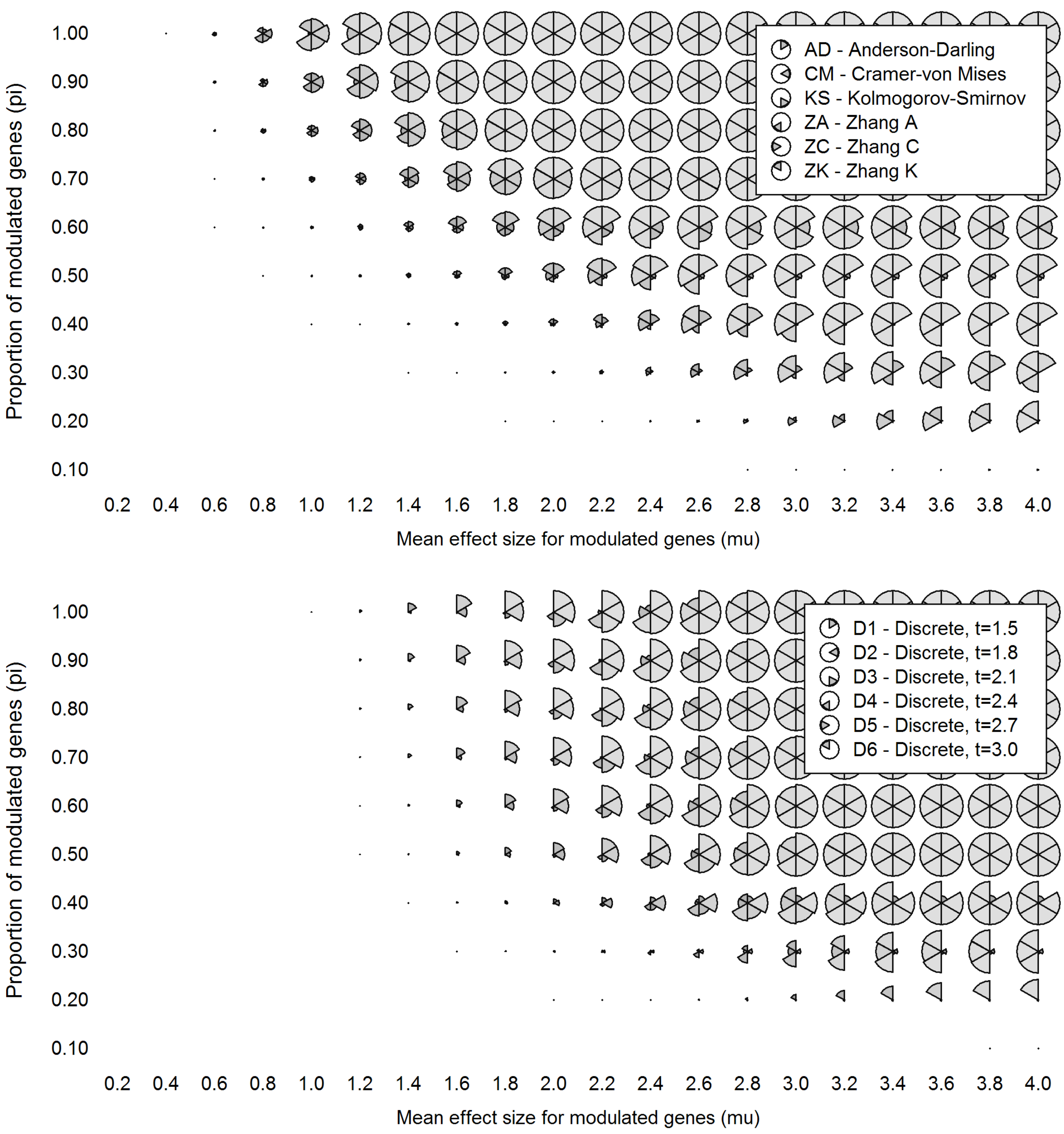

Detection spectrums and overall powers: 30-gene categories, σ=0.5

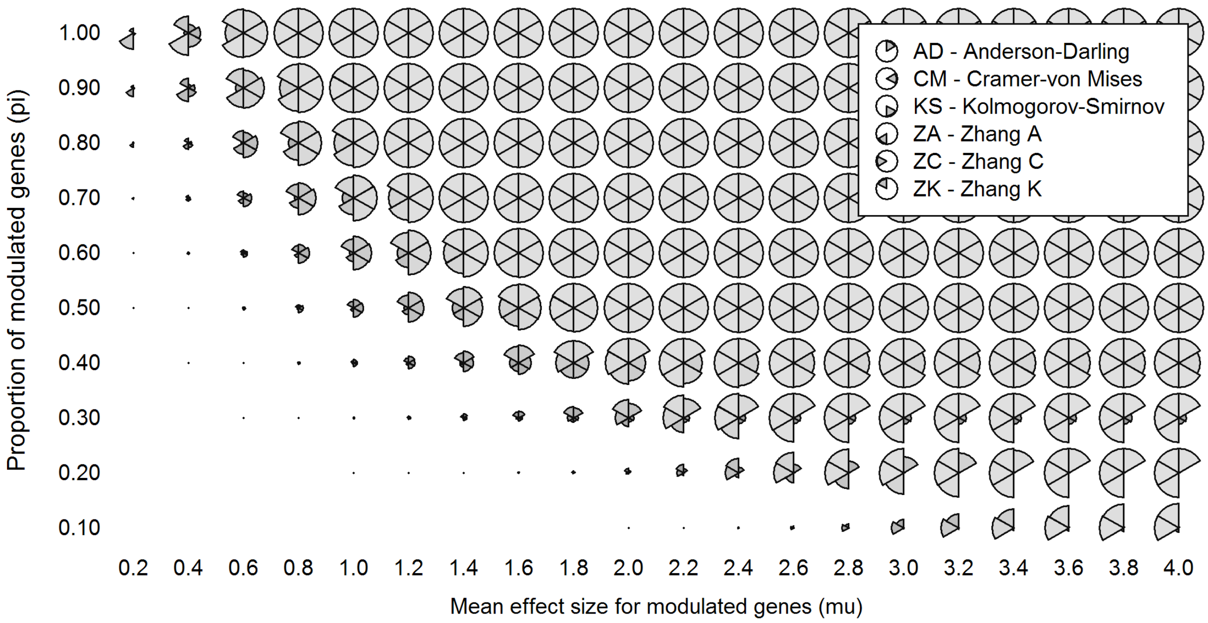

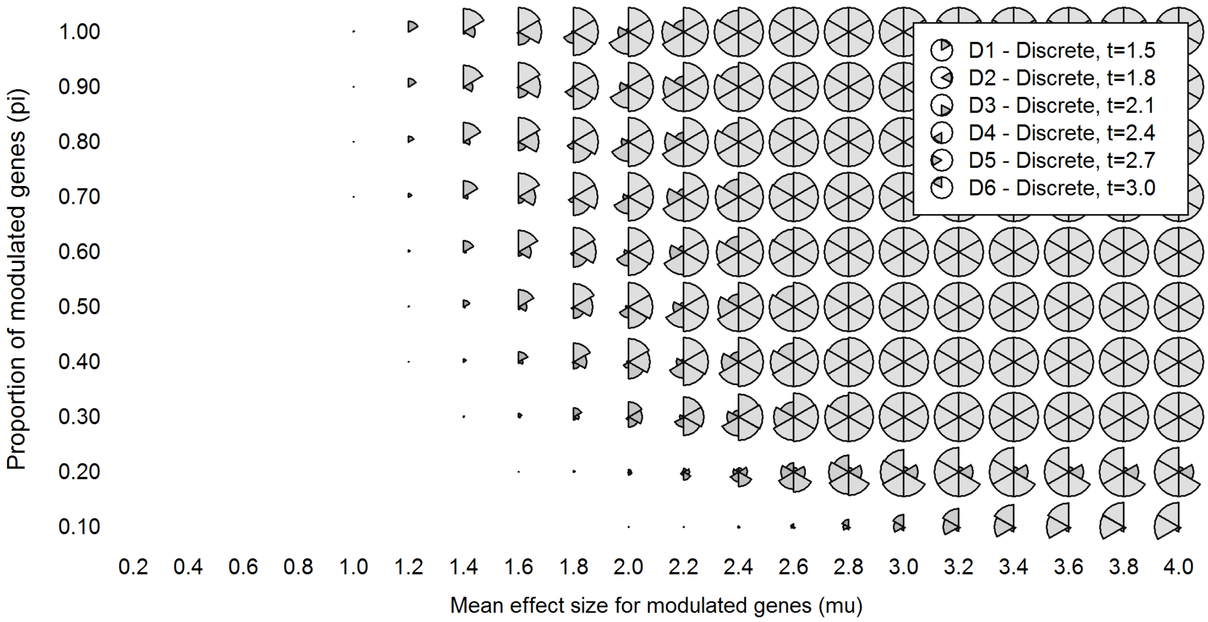

Detection spectrums and overall powers: 100-gene categories, σ=0.5

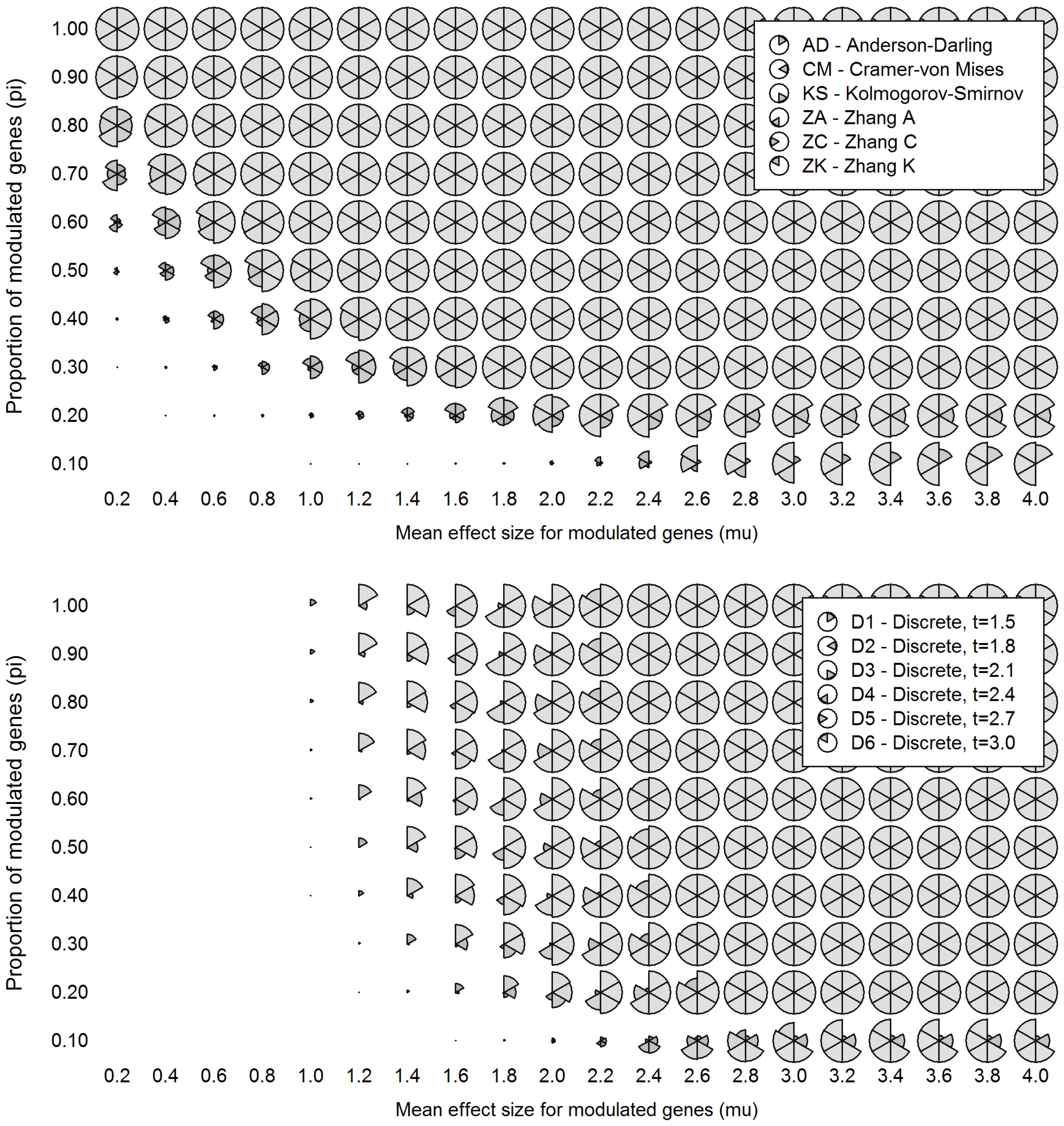

Detection spectrums and overall powers: 10-gene categories, $\sigma = 1.0$

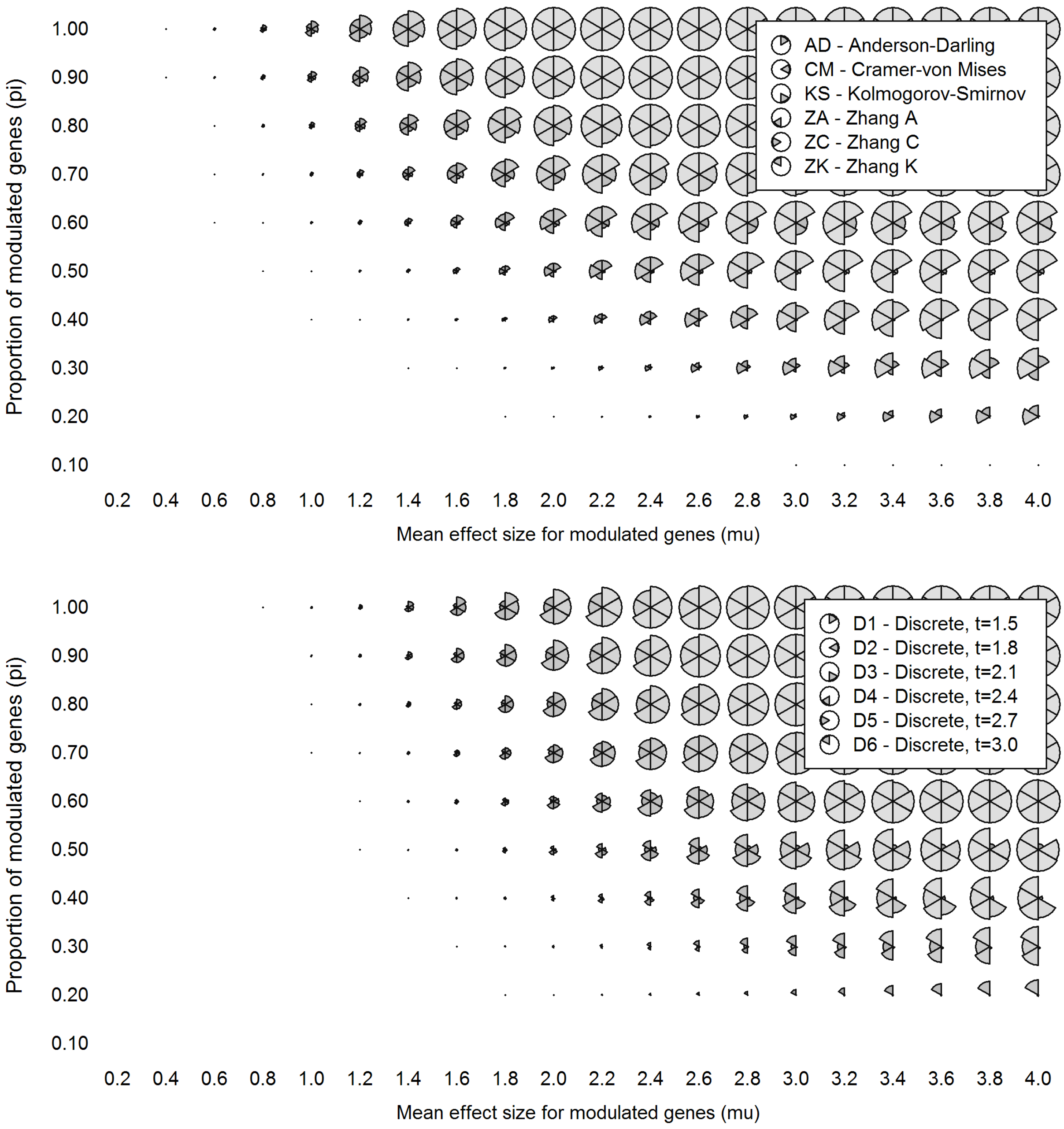

Detection spectrums and overall powers: 30-gene categories, σ=1.0

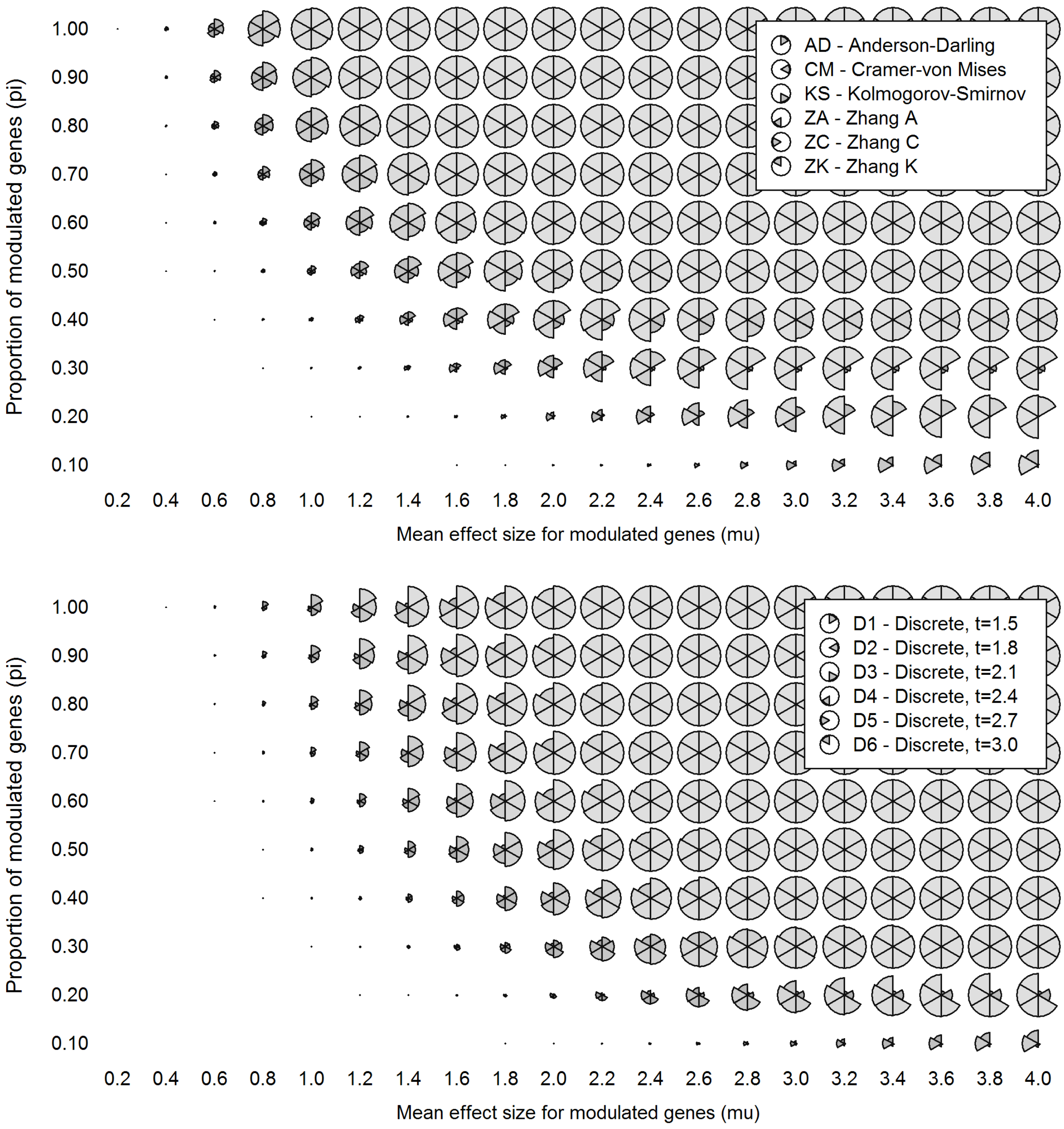

Detection spectrums and overall powers: 100-gene categories, σ=1.0

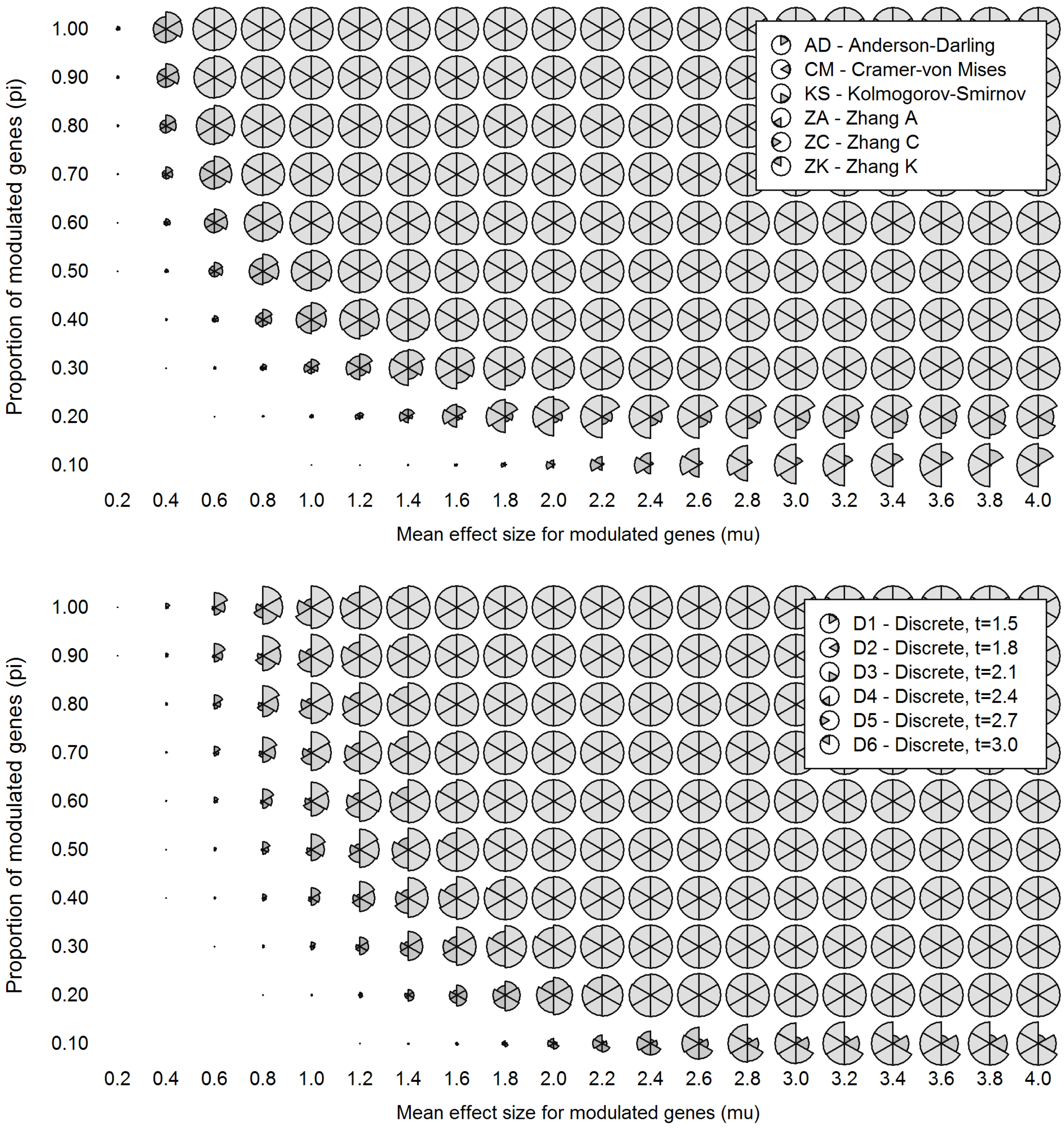

Supplement: Additional data file 1 — A figure representing the complete results of the simulation study. [file gb-2007-8-5-r74-S1.pdf]
