# Supplementary material for: Threshold-free high-power methods for the ontological analysis of genome-wide gene-expression studies
Source: Genome Biol. 2007 May 8;8(5):R74. doi: 10.1186/gb-2007-8-5-r74 (PMC1929143; doi:10.1186/gb-2007-8-5-r74)
Supplement: Additional data file 2 — A table listing overall powers. [file gb-2007-8-5-r74-S2.pdf]

Supplementary Table 1: Overall powers

Effect spread (σ)	All	0.1	0.1	0.1	0.5	0.5	0.5	1.0	1.0	1.0
No. of genes (N)	All	10	30	100	10	30	100	10	30	100
AD - Andersson-Darling	66.5	51.8	73.4	88.5	48.6	66.8	83.2	43.7	63.1	79.1
CM - Cramer-von Mises	58.0	39.7	65.7	84.1	36.2	59.0	79.0	30.7	53.6	73.9
KS - Kolmogorov-Smirnov	58.4	43.1	67.7	86.1	36.2	59.4	80.0	29.1	51.6	72.0
ZA - Zhang A	64.8	49.5	70.7	86.5	45.7	65.8	82.7	41.2	62.3	79.4
ZC - Zhang C	66.5	49.0	71.8	86.7	48.3	66.2	82.3	46.8	67.0	80.8
ZK - Zhang K	69.9	59.6	80.3	91.8	51.5	70.6	85.5	44.8	64.6	80.0
D1 - Discrete, t=1.5	51.7	34.3	53.2	61.6	39.2	54.2	66.0	27.5	55.0	74.1
D2 - Discrete, t=1.8	51.9	41.7	50.5	58.5	40.8	52.5	63.5	30.5	55.5	74.2
D3 - Discrete, t=2.1	49.7	35.4	46.3	52.6	34.6	51.6	61.0	33.6	57.8	74.7
D4 - Discrete, t=2.4	47.2	34.8	40.9	44.8	35.8	47.3	57.0	37.5	55.5	71.4
D5 - Discrete, t=2.7	43.4	32.6	37.2	38.8	29.5	45.0	50.7	31.3	55.2	70.1
D6 - Discrete, t=3.0	39.3	26.1	30.0	30.2	30.4	41.9	44.8	36.3	47.7	66.6

This table lists the overall (average) powers (%) across all four-parameter configurations (first column) and, to allow more detailed study, for specific values of σ and N (second to tenth column).
