# Supplementary material for: Threshold-free high-power methods for the ontological analysis of genome-wide gene-expression studies
Source: Genome Biol. 2007 May 8;8(5):R74. doi: 10.1186/gb-2007-8-5-r74 (PMC1929143; doi:10.1186/gb-2007-8-5-r74)
Supplement: Additional data file 3 — A table containing the complete data from the method-method agreement assessment study. [file gb-2007-8-5-r74-S3.pdf]

Supplementary Table 2

This table represents the complete data from the method-method agreement assessment study. The first table lists the agreements across all simulations (underlies the multi-dimensional scaling plots given in the main paper). The subsequent tables lists the results π and μ for specific values of N and σ (yielded similar multi-dimensional scalings; not shown). To facilitate reading, the tables have been arranged by σ value, such that the results obtained with $\sigma = 0.1$ are listed first, the results for $\sigma = 0.5$ second, and the results for $\sigma = 1.0$ last.

All simulations

**Jaccard distance**	AD	CM	KS	ZA	ZC	ZK	D1	D2	D3	D4	D5	D6
AD - Andersson-Darling	·	0.91	0.90	0.95	0.93	0.94	0.84	0.83	0.80	0.77	0.72	0.68
CM - Cramer-von Mises	0.91	·	0.96	0.89	0.85	0.86	0.84	0.79	0.75	0.71	0.68	0.64
KS - Kolmogorov-Smirnov	0.90	0.96	·	0.89	0.85	0.87	0.84	0.79	0.75	0.71	0.68	0.64
ZA - Zhang A	0.95	0.89	0.89	·	0.94	0.93	0.84	0.84	0.82	0.79	0.75	0.70
ZC - Zhang C	0.93	0.85	0.85	0.94	·	0.94	0.84	0.84	0.82	0.80	0.76	0.72
ZK - Zhang K	0.94	0.86	0.87	0.93	0.94	·	0.81	0.82	0.79	0.77	0.73	0.69
D1 - Discrete, t=1.5	0.84	0.84	0.84	0.84	0.84	0.81	·	0.93	0.88	0.84	0.80	0.76
D2 - Discrete, t=1.8	0.83	0.79	0.79	0.84	0.84	0.82	0.93	·	0.94	0.89	0.84	0.80
D3 - Discrete, t=2.1	0.80	0.75	0.75	0.82	0.82	0.79	0.88	0.94	·	0.94	0.89	0.84
D4 - Discrete, t=2.4	0.77	0.71	0.71	0.79	0.80	0.77	0.84	0.89	0.94	·	0.93	0.89
D5 - Discrete, t=2.7	0.72	0.68	0.68	0.75	0.76	0.73	0.80	0.84	0.89	0.93	·	0.93
D6 - Discrete, t=3.0	0.68	0.64	0.64	0.70	0.72	0.69	0.76	0.80	0.84	0.89	0.93	·

**Spearman correlation**	AD	CM	KS	ZA	ZC	ZK	D1	D2	D3	D4	D5	D6
AD - Andersson-Darling	·	0.94	0.94	0.98	0.97	0.98	0.84	0.78	0.73	0.67	0.63	0.59
CM - Cramer-von Mises	0.94	·	0.99	0.94	0.84	0.87	0.73	0.64	0.56	0.48	0.43	0.39
KS - Kolmogorov-Smirnov	0.94	0.99	·	0.94	0.85	0.89	0.73	0.64	0.57	0.49	0.44	0.40
ZA - Zhang A	0.98	0.94	0.94	·	0.96	0.97	0.80	0.75	0.72	0.67	0.63	0.60
ZC - Zhang C	0.97	0.84	0.85	0.96	·	0.99	0.83	0.82	0.80	0.77	0.75	0.72
ZK - Zhang K	0.98	0.87	0.89	0.97	0.99	·	0.83	0.80	0.77	0.73	0.70	0.67
D1 - Discrete, t=1.5	0.84	0.73	0.73	0.80	0.83	0.83	·	0.90	0.81	0.70	0.63	0.58
D2 - Discrete, t=1.8	0.78	0.64	0.64	0.75	0.82	0.80	0.90	·	0.90	0.80	0.73	0.68
D3 - Discrete, t=2.1	0.73	0.56	0.57	0.72	0.80	0.77	0.81	0.90	·	0.89	0.83	0.78
D4 - Discrete, t=2.4	0.67	0.48	0.49	0.67	0.77	0.73	0.70	0.80	0.89	·	0.92	0.86
D5 - Discrete, t=2.7	0.63	0.43	0.44	0.63	0.75	0.70	0.63	0.73	0.83	0.92	·	0.92
D6 - Discrete, t=3.0	0.59	0.39	0.40	0.60	0.72	0.67	0.58	0.68	0.78	0.86	0.92	·

N=10, σ=0.1

**Jaccard distance**	AD	CM	KS	ZA	ZC	ZK	D1	D2	D3	D4	D5	D6
AD - Andersson-Darling	·	0.85	0.84	0.93	0.91	0.92	0.82	0.86	0.82	0.79	0.72	0.68
CM - Cramer-von Mises	0.85	·	0.94	0.86	0.79	0.79	0.87	0.77	0.75	0.70	0.65	0.64
KS - Kolmogorov-Smirnov	0.84	0.94	·	0.86	0.78	0.82	0.88	0.76	0.74	0.69	0.63	0.62
ZA - Zhang A	0.93	0.86	0.86	·	0.90	0.88	0.82	0.84	0.82	0.80	0.73	0.70
ZC - Zhang C	0.91	0.79	0.78	0.90	·	0.89	0.83	0.88	0.85	0.84	0.81	0.76
ZK - Zhang K	0.92	0.79	0.82	0.88	0.89	·	0.75	0.81	0.76	0.75	0.71	0.66
D1 - Discrete, t=1.5	0.82	0.87	0.88	0.82	0.83	0.75	·	0.86	0.84	0.78	0.73	0.72
D2 - Discrete, t=1.8	0.86	0.77	0.76	0.84	0.88	0.81	0.86	·	0.94	0.86	0.79	0.75
D3 - Discrete, t=2.1	0.82	0.75	0.74	0.82	0.85	0.76	0.84	0.94	·	0.92	0.84	0.81
D4 - Discrete, t=2.4	0.79	0.70	0.69	0.80	0.84	0.75	0.78	0.86	0.92	·	0.91	0.86
D5 - Discrete, t=2.7	0.72	0.65	0.63	0.73	0.81	0.71	0.73	0.79	0.84	0.91	·	0.93
D6 - Discrete, t=3.0	0.68	0.64	0.62	0.70	0.76	0.66	0.72	0.75	0.81	0.86	0.93	·

**Spearman correlation**	AD	CM	KS	ZA	ZC	ZK	D1	D2	D3	D4	D5	D6
AD - Andersson-Darling	·	0.92	0.90	0.98	0.96	0.99	0.80	0.73	0.66	0.56	0.52	0.49
CM - Cramer-von Mises	0.92	·	0.98	0.92	0.80	0.86	0.65	0.53	0.44	0.31	0.26	0.22
KS - Kolmogorov-Smirnov	0.90	0.98	·	0.91	0.78	0.86	0.62	0.51	0.42	0.30	0.24	0.20
ZA - Zhang A	0.98	0.92	0.91	·	0.96	0.97	0.75	0.70	0.64	0.55	0.51	0.48
ZC - Zhang C	0.96	0.80	0.78	0.96	·	0.98	0.79	0.77	0.74	0.68	0.66	0.64
ZK - Zhang K	0.99	0.86	0.86	0.97	0.98	·	0.80	0.75	0.69	0.61	0.58	0.55
D1 - Discrete, t=1.5	0.80	0.65	0.62	0.75	0.79	0.80	·	0.87	0.69	0.54	0.51	0.48
D2 - Discrete, t=1.8	0.73	0.53	0.51	0.70	0.77	0.75	0.87	·	0.86	0.74	0.71	0.69
D3 - Discrete, t=2.1	0.66	0.44	0.42	0.64	0.74	0.69	0.69	0.86	·	0.90	0.87	0.85
D4 - Discrete, t=2.4	0.56	0.31	0.30	0.55	0.68	0.61	0.54	0.74	0.90	·	0.96	0.92
D5 - Discrete, t=2.7	0.52	0.26	0.24	0.51	0.66	0.58	0.51	0.71	0.87	0.96	·	0.96
D6 - Discrete, t=3.0	0.49	0.22	0.20	0.48	0.64	0.55	0.48	0.69	0.85	0.92	0.96	·

N=30, σ=0.1

**Jaccard distance**	AD	CM	KS	ZA	ZC	ZK	D1	D2	D3	D4	D5	D6
AD - Andersson-Darling	·	0.90	0.89	0.95	0.92	0.93	0.78	0.75	0.70	0.64	0.57	0.51
CM - Cramer-von Mises	0.90	·	0.96	0.89	0.84	0.85	0.74	0.69	0.62	0.59	0.52	0.48
KS - Kolmogorov-Smirnov	0.89	0.96	·	0.87	0.82	0.86	0.73	0.68	0.61	0.57	0.51	0.47
ZA - Zhang A	0.95	0.89	0.87	·	0.93	0.90	0.78	0.76	0.72	0.67	0.60	0.54
ZC - Zhang C	0.92	0.84	0.82	0.93	·	0.91	0.78	0.77	0.73	0.68	0.64	0.58
ZK - Zhang K	0.93	0.85	0.86	0.90	0.91	·	0.73	0.70	0.66	0.60	0.56	0.50
D1 - Discrete, t=1.5	0.78	0.74	0.73	0.78	0.78	0.73	·	0.93	0.85	0.79	0.72	0.67
D2 - Discrete, t=1.8	0.75	0.69	0.68	0.76	0.77	0.70	0.93	·	0.91	0.85	0.77	0.72
D3 - Discrete, t=2.1	0.70	0.62	0.61	0.72	0.73	0.66	0.85	0.91	·	0.93	0.85	0.79
D4 - Discrete, t=2.4	0.64	0.59	0.57	0.67	0.68	0.60	0.79	0.85	0.93	·	0.91	0.85
D5 - Discrete, t=2.7	0.57	0.52	0.51	0.60	0.64	0.56	0.72	0.77	0.85	0.91	·	0.93
D6 - Discrete, t=3.0	0.51	0.48	0.47	0.54	0.58	0.50	0.67	0.72	0.79	0.85	0.93	·

**Spearman correlation**	AD	CM	KS	ZA	ZC	ZK	D1	D2	D3	D4	D5	D6
AD - Andersson-Darling	·	0.92	0.91	0.97	0.96	0.98	0.77	0.71	0.63	0.52	0.48	0.46
CM - Cramer-von Mises	0.92	·	0.99	0.93	0.79	0.85	0.63	0.51	0.38	0.27	0.21	0.18
KS - Kolmogorov-Smirnov	0.91	0.99	·	0.92	0.78	0.85	0.60	0.48	0.36	0.26	0.20	0.17
ZA - Zhang A	0.97	0.93	0.92	·	0.94	0.96	0.70	0.64	0.58	0.49	0.46	0.45
ZC - Zhang C	0.96	0.79	0.78	0.94	·	0.99	0.77	0.76	0.72	0.65	0.64	0.64
ZK - Zhang K	0.98	0.85	0.85	0.96	0.99	·	0.78	0.74	0.68	0.59	0.57	0.56
D1 - Discrete, t=1.5	0.77	0.63	0.60	0.70	0.77	0.78	·	0.86	0.67	0.47	0.44	0.43
D2 - Discrete, t=1.8	0.71	0.51	0.48	0.64	0.76	0.74	0.86	·	0.86	0.68	0.64	0.63
D3 - Discrete, t=2.1	0.63	0.38	0.36	0.58	0.72	0.68	0.67	0.86	·	0.83	0.80	0.81
D4 - Discrete, t=2.4	0.52	0.27	0.26	0.49	0.65	0.59	0.47	0.68	0.83	·	0.93	0.87
D5 - Discrete, t=2.7	0.48	0.21	0.20	0.46	0.64	0.57	0.44	0.64	0.80	0.93	·	0.93
D6 - Discrete, t=3.0	0.46	0.18	0.17	0.45	0.64	0.56	0.43	0.63	0.81	0.87	0.93	·

N=100, σ=0.1

**Jaccard distance**	AD	CM	KS	ZA	ZC	ZK	D1	D2	D3	D4	D5	D6
AD - Andersson-Darling	·	0.95	0.95	0.96	0.95	0.96	0.71	0.66	0.60	0.53	0.48	0.41
CM - Cramer-von Mises	0.95	·	0.97	0.92	0.91	0.92	0.69	0.62	0.56	0.50	0.46	0.39
KS - Kolmogorov-Smirnov	0.95	0.97	·	0.93	0.91	0.93	0.69	0.63	0.57	0.50	0.46	0.38
ZA - Zhang A	0.96	0.92	0.93	·	0.98	0.94	0.72	0.69	0.63	0.57	0.52	0.44
ZC - Zhang C	0.95	0.91	0.91	0.98	·	0.95	0.73	0.70	0.65	0.58	0.52	0.44
ZK - Zhang K	0.96	0.92	0.93	0.94	0.95	·	0.70	0.66	0.61	0.53	0.47	0.38
D1 - Discrete, t=1.5	0.71	0.69	0.69	0.72	0.73	0.70	·	0.92	0.85	0.78	0.73	0.65
D2 - Discrete, t=1.8	0.66	0.62	0.63	0.69	0.70	0.66	0.92	·	0.93	0.85	0.79	0.71
D3 - Discrete, t=2.1	0.60	0.56	0.57	0.63	0.65	0.61	0.85	0.93	·	0.92	0.86	0.78
D4 - Discrete, t=2.4	0.53	0.50	0.50	0.57	0.58	0.53	0.78	0.85	0.92	·	0.94	0.85
D5 - Discrete, t=2.7	0.48	0.46	0.46	0.52	0.52	0.47	0.73	0.79	0.86	0.94	·	0.91
D6 - Discrete, t=3.0	0.41	0.39	0.38	0.44	0.44	0.38	0.65	0.71	0.78	0.85	0.91	·

**Spearman correlation**	AD	CM	KS	ZA	ZC	ZK	D1	D2	D3	D4	D5	D6
AD - Andersson-Darling	·	0.93	0.92	0.97	0.96	0.98	0.62	0.50	0.44	0.36	0.36	0.38
CM - Cramer-von Mises	0.93	·	0.99	0.93	0.79	0.86	0.40	0.23	0.14	0.07	0.07	0.09
KS - Kolmogorov-Smirnov	0.92	0.99	·	0.92	0.78	0.85	0.37	0.20	0.12	0.05	0.06	0.08
ZA - Zhang A	0.97	0.93	0.92	·	0.94	0.96	0.50	0.41	0.37	0.32	0.34	0.36
ZC - Zhang C	0.96	0.79	0.78	0.94	·	0.99	0.67	0.61	0.60	0.54	0.55	0.57
ZK - Zhang K	0.98	0.86	0.85	0.96	0.99	·	0.65	0.57	0.54	0.47	0.47	0.49
D1 - Discrete, t=1.5	0.62	0.40	0.37	0.50	0.67	0.65	·	0.81	0.67	0.46	0.35	0.39
D2 - Discrete, t=1.8	0.50	0.23	0.20	0.41	0.61	0.57	0.81	·	0.80	0.62	0.51	0.53
D3 - Discrete, t=2.1	0.44	0.14	0.12	0.37	0.60	0.54	0.67	0.80	·	0.81	0.71	0.73
D4 - Discrete, t=2.4	0.36	0.07	0.05	0.32	0.54	0.47	0.46	0.62	0.81	·	0.87	0.83
D5 - Discrete, t=2.7	0.36	0.07	0.06	0.34	0.55	0.47	0.35	0.51	0.71	0.87	·	0.89
D6 - Discrete, t=3.0	0.38	0.09	0.08	0.36	0.57	0.49	0.39	0.53	0.73	0.83	0.89	·

N=10, σ=0.5

**Jaccard distance**	AD	CM	KS	ZA	ZC	ZK	D1	D2	D3	D4	D5	D6
AD - Andersson-Darling	·	0.86	0.86	0.96	0.91	0.94	0.89	0.90	0.85	0.84	0.79	0.76
CM - Cramer-von Mises	0.86	·	0.95	0.88	0.80	0.82	0.86	0.81	0.80	0.76	0.75	0.71
KS - Kolmogorov-Smirnov	0.86	0.95	·	0.88	0.81	0.83	0.87	0.82	0.82	0.77	0.76	0.73
ZA - Zhang A	0.96	0.88	0.88	·	0.92	0.92	0.89	0.90	0.87	0.85	0.82	0.79
ZC - Zhang C	0.91	0.80	0.81	0.92	·	0.94	0.88	0.90	0.86	0.87	0.81	0.82
ZK - Zhang K	0.94	0.82	0.83	0.92	0.94	·	0.87	0.88	0.83	0.84	0.78	0.78
D1 - Discrete, t=1.5	0.89	0.86	0.87	0.89	0.88	0.87	·	0.93	0.90	0.85	0.81	0.78
D2 - Discrete, t=1.8	0.90	0.81	0.82	0.90	0.90	0.88	0.93	·	0.94	0.89	0.85	0.81
D3 - Discrete, t=2.1	0.85	0.80	0.82	0.87	0.86	0.83	0.90	0.94	·	0.94	0.90	0.86
D4 - Discrete, t=2.4	0.84	0.76	0.77	0.85	0.87	0.84	0.85	0.89	0.94	·	0.94	0.90
D5 - Discrete, t=2.7	0.79	0.75	0.76	0.82	0.81	0.78	0.81	0.85	0.90	0.94	·	0.94
D6 - Discrete, t=3.0	0.76	0.71	0.73	0.79	0.82	0.78	0.78	0.81	0.86	0.90	0.94	·

**Spearman correlation**	AD	CM	KS	ZA	ZC	ZK	D1	D2	D3	D4	D5	D6
AD - Andersson-Darling	·	0.94	0.94	0.99	0.97	0.98	0.90	0.86	0.80	0.72	0.63	0.56
CM - Cramer-von Mises	0.94	·	0.99	0.93	0.84	0.88	0.80	0.71	0.63	0.53	0.43	0.35
KS - Kolmogorov-Smirnov	0.94	0.99	·	0.93	0.85	0.89	0.80	0.72	0.64	0.55	0.44	0.36
ZA - Zhang A	0.99	0.93	0.93	·	0.97	0.97	0.87	0.84	0.79	0.72	0.63	0.56
ZC - Zhang C	0.97	0.84	0.85	0.97	·	0.98	0.88	0.88	0.86	0.81	0.74	0.68
ZK - Zhang K	0.98	0.88	0.89	0.97	0.98	·	0.89	0.86	0.83	0.77	0.69	0.63
D1 - Discrete, t=1.5	0.90	0.80	0.80	0.87	0.88	0.89	·	0.93	0.85	0.74	0.62	0.55
D2 - Discrete, t=1.8	0.86	0.71	0.72	0.84	0.88	0.86	0.93	·	0.92	0.82	0.72	0.65
D3 - Discrete, t=2.1	0.80	0.63	0.64	0.79	0.86	0.83	0.85	0.92	·	0.91	0.81	0.76
D4 - Discrete, t=2.4	0.72	0.53	0.55	0.72	0.81	0.77	0.74	0.82	0.91	·	0.91	0.85
D5 - Discrete, t=2.7	0.63	0.43	0.44	0.63	0.74	0.69	0.62	0.72	0.81	0.91	·	0.93
D6 - Discrete, t=3.0	0.56	0.35	0.36	0.56	0.68	0.63	0.55	0.65	0.76	0.85	0.93	·

N=30, σ=0.5

**Jaccard distance**	AD	CM	KS	ZA	ZC	ZK	D1	D2	D3	D4	D5	D6
AD - Andersson-Darling	·	0.91	0.90	0.94	0.92	0.94	0.87	0.84	0.82	0.77	0.72	0.68
CM - Cramer-von Mises	0.91	·	0.96	0.88	0.84	0.86	0.83	0.79	0.74	0.71	0.66	0.64
KS - Kolmogorov-Smirnov	0.90	0.96	·	0.88	0.84	0.87	0.84	0.79	0.75	0.71	0.67	0.64
ZA - Zhang A	0.94	0.88	0.88	·	0.93	0.92	0.86	0.85	0.83	0.79	0.74	0.71
ZC - Zhang C	0.92	0.84	0.84	0.93	·	0.94	0.87	0.86	0.85	0.81	0.79	0.75
ZK - Zhang K	0.94	0.86	0.87	0.92	0.94	·	0.83	0.82	0.80	0.76	0.74	0.70
D1 - Discrete, t=1.5	0.87	0.83	0.84	0.86	0.87	0.83	·	0.94	0.89	0.85	0.80	0.76
D2 - Discrete, t=1.8	0.84	0.79	0.79	0.85	0.86	0.82	0.94	·	0.94	0.90	0.84	0.81
D3 - Discrete, t=2.1	0.82	0.74	0.75	0.83	0.85	0.80	0.89	0.94	·	0.95	0.89	0.85
D4 - Discrete, t=2.4	0.77	0.71	0.71	0.79	0.81	0.76	0.85	0.90	0.95	·	0.93	0.90
D5 - Discrete, t=2.7	0.72	0.66	0.67	0.74	0.79	0.74	0.80	0.84	0.89	0.93	·	0.95
D6 - Discrete, t=3.0	0.68	0.64	0.64	0.71	0.75	0.70	0.76	0.81	0.85	0.90	0.95	·

**Spearman correlation**	AD	CM	KS	ZA	ZC	ZK	D1	D2	D3	D4	D5	D6
AD - Andersson-Darling	·	0.94	0.95	0.98	0.96	0.98	0.91	0.87	0.82	0.76	0.69	0.59
CM - Cramer-von Mises	0.94	·	0.99	0.93	0.83	0.87	0.83	0.74	0.66	0.57	0.48	0.37
KS - Kolmogorov-Smirnov	0.95	0.99	·	0.94	0.85	0.89	0.84	0.76	0.68	0.59	0.51	0.40
ZA - Zhang A	0.98	0.93	0.94	·	0.96	0.97	0.88	0.84	0.80	0.75	0.69	0.60
ZC - Zhang C	0.96	0.83	0.85	0.96	·	0.99	0.89	0.88	0.87	0.85	0.80	0.73
ZK - Zhang K	0.98	0.87	0.89	0.97	0.99	·	0.90	0.87	0.85	0.81	0.76	0.68
D1 - Discrete, t=1.5	0.91	0.83	0.84	0.88	0.89	0.90	·	0.95	0.89	0.79	0.68	0.56
D2 - Discrete, t=1.8	0.87	0.74	0.76	0.84	0.88	0.87	0.95	·	0.94	0.86	0.76	0.65
D3 - Discrete, t=2.1	0.82	0.66	0.68	0.80	0.87	0.85	0.89	0.94	·	0.92	0.83	0.73
D4 - Discrete, t=2.4	0.76	0.57	0.59	0.75	0.85	0.81	0.79	0.86	0.92	·	0.91	0.82
D5 - Discrete, t=2.7	0.69	0.48	0.51	0.69	0.80	0.76	0.68	0.76	0.83	0.91	·	0.91
D6 - Discrete, t=3.0	0.59	0.37	0.40	0.60	0.73	0.68	0.56	0.65	0.73	0.82	0.91	·

N=100, σ=0.5

**Jaccard distance**	AD	CM	KS	ZA	ZC	ZK	D1	D2	D3	D4	D5	D6
AD - Andersson-Darling	·	0.95	0.95	0.95	0.95	0.96	0.82	0.78	0.73	0.69	0.64	0.59
CM - Cramer-von Mises	0.95	·	0.97	0.91	0.90	0.92	0.80	0.75	0.70	0.66	0.61	0.57
KS - Kolmogorov-Smirnov	0.95	0.97	·	0.92	0.91	0.93	0.81	0.76	0.70	0.67	0.62	0.58
ZA - Zhang A	0.95	0.91	0.92	·	0.98	0.96	0.82	0.79	0.76	0.73	0.67	0.62
ZC - Zhang C	0.95	0.90	0.91	0.98	·	0.96	0.83	0.81	0.78	0.74	0.68	0.63
ZK - Zhang K	0.96	0.92	0.93	0.96	0.96	·	0.80	0.78	0.75	0.71	0.65	0.59
D1 - Discrete, t=1.5	0.82	0.80	0.81	0.82	0.83	0.80	·	0.94	0.88	0.84	0.79	0.74
D2 - Discrete, t=1.8	0.78	0.75	0.76	0.79	0.81	0.78	0.94	·	0.94	0.90	0.84	0.79
D3 - Discrete, t=2.1	0.73	0.70	0.70	0.76	0.78	0.75	0.88	0.94	·	0.95	0.90	0.84
D4 - Discrete, t=2.4	0.69	0.66	0.67	0.73	0.74	0.71	0.84	0.90	0.95	·	0.94	0.88
D5 - Discrete, t=2.7	0.64	0.61	0.62	0.67	0.68	0.65	0.79	0.84	0.90	0.94	·	0.94
D6 - Discrete, t=3.0	0.59	0.57	0.58	0.62	0.63	0.59	0.74	0.79	0.84	0.88	0.94	·

**Spearman correlation**	AD	CM	KS	ZA	ZC	ZK	D1	D2	D3	D4	D5	D6
AD - Andersson-Darling	·	0.95	0.97	0.98	0.97	0.98	0.80	0.70	0.66	0.62	0.60	0.56
CM - Cramer-von Mises	0.95	·	1.00	0.95	0.85	0.89	0.71	0.56	0.50	0.43	0.40	0.36
KS - Kolmogorov-Smirnov	0.97	1.00	·	0.96	0.88	0.91	0.72	0.59	0.53	0.46	0.43	0.40
ZA - Zhang A	0.98	0.95	0.96	·	0.96	0.98	0.76	0.67	0.64	0.60	0.59	0.57
ZC - Zhang C	0.97	0.85	0.88	0.96	·	0.99	0.80	0.75	0.75	0.73	0.73	0.71
ZK - Zhang K	0.98	0.89	0.91	0.98	0.99	·	0.80	0.74	0.72	0.70	0.69	0.67
D1 - Discrete, t=1.5	0.80	0.71	0.72	0.76	0.80	0.80	·	0.89	0.81	0.72	0.64	0.52
D2 - Discrete, t=1.8	0.70	0.56	0.59	0.67	0.75	0.74	0.89	·	0.90	0.82	0.72	0.60
D3 - Discrete, t=2.1	0.66	0.50	0.53	0.64	0.75	0.72	0.81	0.90	·	0.89	0.81	0.69
D4 - Discrete, t=2.4	0.62	0.43	0.46	0.60	0.73	0.70	0.72	0.82	0.89	·	0.90	0.78
D5 - Discrete, t=2.7	0.60	0.40	0.43	0.59	0.73	0.69	0.64	0.72	0.81	0.90	·	0.88
D6 - Discrete, t=3.0	0.56	0.36	0.40	0.57	0.71	0.67	0.52	0.60	0.69	0.78	0.88	·

N=10, σ=1.0

**Jaccard distance**	AD	CM	KS	ZA	ZC	ZK	D1	D2	D3	D4	D5	D6
AD - Andersson-Darling	·	0.87	0.85	0.96	0.92	0.94	0.84	0.87	0.89	0.89	0.85	0.84
CM - Cramer-von Mises	0.87	·	0.95	0.89	0.81	0.83	0.91	0.89	0.86	0.82	0.82	0.78
KS - Kolmogorov-Smirnov	0.85	0.95	·	0.87	0.80	0.83	0.94	0.90	0.87	0.83	0.83	0.80
ZA - Zhang A	0.96	0.89	0.87	·	0.92	0.93	0.86	0.89	0.90	0.90	0.87	0.86
ZC - Zhang C	0.92	0.81	0.80	0.92	·	0.95	0.80	0.83	0.86	0.90	0.84	0.88
ZK - Zhang K	0.94	0.83	0.83	0.93	0.95	·	0.83	0.86	0.89	0.91	0.86	0.88
D1 - Discrete, t=1.5	0.84	0.91	0.94	0.86	0.80	0.83	·	0.94	0.90	0.85	0.86	0.82
D2 - Discrete, t=1.8	0.87	0.89	0.90	0.89	0.83	0.86	0.94	·	0.94	0.89	0.89	0.85
D3 - Discrete, t=2.1	0.89	0.86	0.87	0.90	0.86	0.89	0.90	0.94	·	0.93	0.92	0.88
D4 - Discrete, t=2.4	0.89	0.82	0.83	0.90	0.90	0.91	0.85	0.89	0.93	·	0.94	0.91
D5 - Discrete, t=2.7	0.85	0.82	0.83	0.87	0.84	0.86	0.86	0.89	0.92	0.94	·	0.92
D6 - Discrete, t=3.0	0.84	0.78	0.80	0.86	0.88	0.88	0.82	0.85	0.88	0.91	0.92	·

**Spearman correlation**	AD	CM	KS	ZA	ZC	ZK	D1	D2	D3	D4	D5	D6
AD - Andersson-Darling	·	0.95	0.95	0.99	0.97	0.98	0.93	0.92	0.89	0.85	0.79	0.73
CM - Cramer-von Mises	0.95	·	0.99	0.93	0.86	0.88	0.86	0.81	0.76	0.70	0.63	0.58
KS - Kolmogorov-Smirnov	0.95	0.99	·	0.93	0.86	0.89	0.86	0.82	0.77	0.72	0.65	0.60
ZA - Zhang A	0.99	0.93	0.93	·	0.98	0.97	0.92	0.92	0.89	0.86	0.80	0.75
ZC - Zhang C	0.97	0.86	0.86	0.98	·	0.98	0.91	0.92	0.92	0.91	0.87	0.83
ZK - Zhang K	0.98	0.88	0.89	0.97	0.98	·	0.92	0.92	0.90	0.87	0.82	0.78
D1 - Discrete, t=1.5	0.93	0.86	0.86	0.92	0.91	0.92	·	0.94	0.89	0.83	0.76	0.71
D2 - Discrete, t=1.8	0.92	0.81	0.82	0.92	0.92	0.92	0.94	·	0.94	0.88	0.81	0.75
D3 - Discrete, t=2.1	0.89	0.76	0.77	0.89	0.92	0.90	0.89	0.94	·	0.93	0.87	0.81
D4 - Discrete, t=2.4	0.85	0.70	0.72	0.86	0.91	0.87	0.83	0.88	0.93	·	0.92	0.86
D5 - Discrete, t=2.7	0.79	0.63	0.65	0.80	0.87	0.82	0.76	0.81	0.87	0.92	·	0.92
D6 - Discrete, t=3.0	0.73	0.58	0.60	0.75	0.83	0.78	0.71	0.75	0.81	0.86	0.92	·

N=30, σ=1.0

**Jaccard distance**	AD	CM	KS	ZA	ZC	ZK	D1	D2	D3	D4	D5	D6
AD - Andersson-Darling	·	0.90	0.88	0.96	0.92	0.94	0.91	0.91	0.90	0.88	0.84	0.80
CM - Cramer-von Mises	0.90	·	0.96	0.89	0.84	0.86	0.89	0.87	0.84	0.82	0.79	0.77
KS - Kolmogorov-Smirnov	0.88	0.96	·	0.88	0.83	0.86	0.90	0.87	0.84	0.83	0.80	0.78
ZA - Zhang A	0.96	0.89	0.88	·	0.94	0.95	0.91	0.91	0.92	0.90	0.87	0.83
ZC - Zhang C	0.92	0.84	0.83	0.94	·	0.95	0.87	0.88	0.90	0.88	0.88	0.81
ZK - Zhang K	0.94	0.86	0.86	0.95	0.95	·	0.90	0.90	0.92	0.90	0.89	0.83
D1 - Discrete, t=1.5	0.91	0.89	0.90	0.91	0.87	0.90	·	0.95	0.91	0.89	0.86	0.83
D2 - Discrete, t=1.8	0.91	0.87	0.87	0.91	0.88	0.90	0.95	·	0.94	0.92	0.89	0.85
D3 - Discrete, t=2.1	0.90	0.84	0.84	0.92	0.90	0.92	0.91	0.94	·	0.95	0.91	0.87
D4 - Discrete, t=2.4	0.88	0.82	0.83	0.90	0.88	0.90	0.89	0.92	0.95	·	0.94	0.90
D5 - Discrete, t=2.7	0.84	0.79	0.80	0.87	0.88	0.89	0.86	0.89	0.91	0.94	·	0.92
D6 - Discrete, t=3.0	0.80	0.77	0.78	0.83	0.81	0.83	0.83	0.85	0.87	0.90	0.92	·

**Spearman correlation**	AD	CM	KS	ZA	ZC	ZK	D1	D2	D3	D4	D5	D6
AD - Andersson-Darling	·	0.97	0.98	0.99	0.97	0.98	0.97	0.95	0.93	0.90	0.86	0.82
CM - Cramer-von Mises	0.97	·	0.99	0.95	0.90	0.90	0.93	0.89	0.85	0.80	0.75	0.70
KS - Kolmogorov-Smirnov	0.98	0.99	·	0.96	0.91	0.92	0.93	0.90	0.86	0.82	0.77	0.72
ZA - Zhang A	0.99	0.95	0.96	·	0.98	0.98	0.96	0.95	0.93	0.91	0.87	0.84
ZC - Zhang C	0.97	0.90	0.91	0.98	·	0.99	0.94	0.95	0.95	0.94	0.92	0.90
ZK - Zhang K	0.98	0.90	0.92	0.98	0.99	·	0.94	0.95	0.94	0.93	0.90	0.87
D1 - Discrete, t=1.5	0.97	0.93	0.93	0.96	0.94	0.94	·	0.97	0.93	0.90	0.85	0.80
D2 - Discrete, t=1.8	0.95	0.89	0.90	0.95	0.95	0.95	0.97	·	0.96	0.93	0.88	0.84
D3 - Discrete, t=2.1	0.93	0.85	0.86	0.93	0.95	0.94	0.93	0.96	·	0.96	0.91	0.87
D4 - Discrete, t=2.4	0.90	0.80	0.82	0.91	0.94	0.93	0.90	0.93	0.96	·	0.95	0.90
D5 - Discrete, t=2.7	0.86	0.75	0.77	0.87	0.92	0.90	0.85	0.88	0.91	0.95	·	0.94
D6 - Discrete, t=3.0	0.82	0.70	0.72	0.84	0.90	0.87	0.80	0.84	0.87	0.90	0.94	·

N=100, σ=1.0

**Jaccard distance**	AD	CM	KS	ZA	ZC	ZK	D1	D2	D3	D4	D5	D6
AD - Andersson-Darling	·	0.95	0.93	0.96	0.95	0.95	0.93	0.92	0.90	0.87	0.85	0.82
CM - Cramer-von Mises	0.95	·	0.97	0.92	0.90	0.91	0.91	0.88	0.86	0.83	0.82	0.79
KS - Kolmogorov-Smirnov	0.93	0.97	·	0.91	0.89	0.91	0.91	0.89	0.86	0.84	0.83	0.80
ZA - Zhang A	0.96	0.92	0.91	·	0.98	0.97	0.93	0.93	0.93	0.90	0.89	0.86
ZC - Zhang C	0.95	0.90	0.89	0.98	·	0.97	0.92	0.93	0.93	0.90	0.89	0.86
ZK - Zhang K	0.95	0.91	0.91	0.97	0.97	·	0.93	0.93	0.93	0.91	0.89	0.86
D1 - Discrete, t=1.5	0.93	0.91	0.91	0.93	0.92	0.93	·	0.95	0.92	0.90	0.88	0.85
D2 - Discrete, t=1.8	0.92	0.88	0.89	0.93	0.93	0.93	0.95	·	0.95	0.93	0.91	0.88
D3 - Discrete, t=2.1	0.90	0.86	0.86	0.93	0.93	0.93	0.92	0.95	·	0.95	0.93	0.90
D4 - Discrete, t=2.4	0.87	0.83	0.84	0.90	0.90	0.91	0.90	0.93	0.95	·	0.96	0.93
D5 - Discrete, t=2.7	0.85	0.82	0.83	0.89	0.89	0.89	0.88	0.91	0.93	0.96	·	0.95
D6 - Discrete, t=3.0	0.82	0.79	0.80	0.86	0.86	0.86	0.85	0.88	0.90	0.93	0.95	·

**Spearman correlation**	AD	CM	KS	ZA	ZC	ZK	D1	D2	D3	D4	D5	D6
AD - Andersson-Darling	·	0.97	0.98	0.99	0.98	0.97	0.86	0.81	0.78	0.76	0.74	0.74
CM - Cramer-von Mises	0.97	·	0.99	0.96	0.91	0.89	0.83	0.76	0.72	0.68	0.65	0.64
KS - Kolmogorov-Smirnov	0.98	0.99	·	0.97	0.93	0.92	0.84	0.78	0.73	0.70	0.68	0.67
ZA - Zhang A	0.99	0.96	0.97	·	0.98	0.98	0.86	0.82	0.79	0.78	0.77	0.77
ZC - Zhang C	0.98	0.91	0.93	0.98	·	1.00	0.84	0.82	0.81	0.81	0.81	0.82
ZK - Zhang K	0.97	0.89	0.92	0.98	1.00	·	0.84	0.82	0.80	0.80	0.80	0.81
D1 - Discrete, t=1.5	0.86	0.83	0.84	0.86	0.84	0.84	·	0.91	0.87	0.83	0.79	0.76
D2 - Discrete, t=1.8	0.81	0.76	0.78	0.82	0.82	0.82	0.91	·	0.91	0.88	0.85	0.81
D3 - Discrete, t=2.1	0.78	0.72	0.73	0.79	0.81	0.80	0.87	0.91	·	0.90	0.87	0.83
D4 - Discrete, t=2.4	0.76	0.68	0.70	0.78	0.81	0.80	0.83	0.88	0.90	·	0.93	0.89
D5 - Discrete, t=2.7	0.74	0.65	0.68	0.77	0.81	0.80	0.79	0.85	0.87	0.93	·	0.93
D6 - Discrete, t=3.0	0.74	0.64	0.67	0.77	0.82	0.81	0.76	0.81	0.83	0.89	0.93	·
