# Supplementary material for: Threshold-free high-power methods for the ontological analysis of genome-wide gene-expression studies
Source: Genome Biol. 2007 May 8;8(5):R74. doi: 10.1186/gb-2007-8-5-r74 (PMC1929143; doi:10.1186/gb-2007-8-5-r74)
Supplement: Additional data file 4 — A list of additional differential expression studies. [file gb-2007-8-5-r74-S4.pdf]

Supplementary Table 3: Additional differential expression studies

Reference	Conditions compared
Zheng *et al* (2006)	CML, blast crisis vs chronic phase
Radich *et al* (2006)	CML, blast crisis vs chronic phase
	CML, accelerated vs chronic phase
Ross *et al* (2003)	ALL, *BCR/ABL1*-positive vs others
	ALL, *TCF3/RUNX1*-positive vs others
	ALL, *ETV6/RUNX1*-positive vs others
	ALL, *MLL* rearrangement vs others
	ALL, hyperdiploid vs others
	ALL, T-cell type vs B-cell type
Andersson *et al* (2005)	ALL, *TCF3/PBX1*-positive vs others
	ALL, *ETV6/RUNX1*-positive vs others
	ALL, hyperdiploid vs others
	ALL, T-cell type vs B-cell type
Valk *et al* (2004)	AML, normal karyotype vs others
	AML, t(8;21) vs others
	AML, t(15;17) vs others
	AML, inv(16) vs others
	AML, 11q23 rearrangement
	AML, -7 vs others
	AML, +8 vs others
	AML, t(6;9) vs others
Bhattacharjee *et al* (2001)	Lung cancer, adenocarcinoma vs others
	Lung cancer, small cell ca. vs others
	Lung cancer, squamous cell ca. vs others
West *et al* (2001)	Breast cancer, ER-positive vs ER-negative

The full references are given in the bibliography in the main paper.
